# Coordinating ability and versatility of organosulfur-based ligands in transition metal catalyzed hydrogenative and hydrogen auto-transfer processes

**DOI:** 10.3389/fchem.2025.1746644

**Published:** 2026-02-26

**Authors:** Andrei Paraschiv, Daria Brambilla, Giancarlo Cravotto, Cristina Prandi, Salvatore Baldino, Katia Martina

**Affiliations:** 1 Department of Drug Science and Technology, University of Turin, Turin, Italy; 2 Cube Labs Spa, Rome, Italy; 3 Department of Chemistry, NIS Interdepartmental Centre and INSTM Reference Centre, University of Turin, Turin, Italy

**Keywords:** borrowing hydrogen, homogeneous catalysis, hydrogenation, sulfur-based ligands, transfer hydrogenation

## Abstract

Transition metal complexes with sulfur-donor ligands serve as efficient catalysts in numerous homogeneous reactions, owing to the coordination diversity of sulfur-based ligands and the possibility to obtain chiral and achiral complexes. Historically dominated by phosphorus and nitrogen, transition metal compounds with electron-rich sulfur-donor ligands have recently attracted research interest because of their close connection with enzymatic transformation. This review presents a comprehensive overview of recent applications of sulfur-ligated metal complexes in hydrogenative and strictly related hydrogen auto-transfer processes, namely hydrogenation, transfer hydrogenation and borrowing hydrogen reactions. Particular attention is given to the impact of ligand selection on the reaction outcomes, and on the influence of sulfur to give stability at the catalytically active metal complex. Additionally, the review compares conventional hydrogenation and transfer hydrogenation strategies with emerging approaches involving metal-ligand cooperation in the presence of sulfur-based ligands.

## 1 Introduction

The development of powerful and selective catalysts has been a major focus of research driven by the demand for green and sustainable chemical processes, which are essential for the selective formation of products (Anastas et al., 2001; Yenare et al., 2025).

Catalytic hydrogenation (HY) represents a foundational process within the domain of organic synthesis, enabling the straightforward conversion of multiple double bonds (e.g., C=C, C=N, C=O) to their reduced forms. This method has found extensive industrial application in numerous contexts. These processes are employed in a variety of fields, including fine chemicals and pharmaceuticals synthesis (De Vries and Elsevier, 2007). The reductive transformations are performed through two distinct strategies: direct HY, which involves the use of pressurized hydrogen gas, and transfer hydrogenation (TH), which is characterized by the transfer of hydrogen atoms from one molecule to another (Wang and Astruc, 2015). The latter reaction, which refers to the reduction of a molecule from a H_2_-source, is a convenient, powerful and attractive alternative to direct HY. The reasons for this include the fact that it does not require hazardous pressurized H_2_ gas or elaborate experimental setups; the hydrogen donors are readily available, cheap and safe; the major side products are easily recycled; and the used catalysts are readily accessible and stable in aerobic conditions (Wang and Astruc, 2015).

Another elegant and relatively recent application of transition metal catalysis for implementing highly important organic transformations is the so-called “borrowing hydrogen” (BH) approach (also known as hydrogen auto-transfer). BH initially involves two compounds which are not normally reactive towards each other (e.g., alcohol and amine). One H_2_ molecule is then extracted (“borrowed”) from the alcohol forming a reactive intermediate and, after the desired process takes place (generally a condensation), it is given back to the obtained product (Guillena et al., 2007). As a matter of fact, BH may be considered as a cascade or domino dehydrogenation (DHY)/condensation/TH reactions, it does not occur spontaneously and needs the employment of transition metal catalysts to mediate the H_2_ “shuttle”, particularly for the generation of the reactive intermediate by DHY which is thermodynamically disfavored and requires a high activation energy (Murzin, 2015).

In (de)hydrogenative transformations, transition metal complexes based on 4d (e.g., Ru, Rh, Pd) and 5d metals (e.g., Re, Os, Ir, Pt)—particularly ruthenium—are most commonly used (Pritchard et al., 2015; Singh et al., 2023). However, their scarcity, high cost, and toxicity have driven interest toward more sustainable alternatives (Filonenko et al., 2018). As a result, recent efforts focus on developing 3d (first-row transition) metal complexes for industrially relevant redox processes (Wen et al., 2021; Zell and Langer, 2018). Another important field of research is the study of new metallic complexes, because the alteration of the orbital structure of the metal has a direct effect on the activation energy of the steps involved in the catalytic process. Ligands can influence the stereo-, regio- and chemoselectivity of metallic catalysts (Handa, 2024). Furthermore, they have been shown to improve the solubility of said catalysts in organic solvents, whilst also extending their stability and lifetime (Berrisford et al., 2003). Finally, it has been determined that the ligand can affect the operational conditions of the catalyst, including compatibility with air and/or moisture, reaction temperature and pressure. In the domain of asymmetric catalysis, the design of ligands assumes paramount importance. The employment of a catalyst endowed with an enantiopure chiral ligand facilitates the selective formation of one of the possible enantiomers and numerous exemplars of considerable pertinence can be ascertained in the extant literature (Gladiali and Alberico, 2006; Zhang W. et al., 2007).

A potential disadvantage of catalytic systems is that they generally contain phosphines in the ligand backbone. Phosphines exhibit numerous limitations, including low air, humidity and thermal stability, as well as high synthesis costs, which undermines their versatility and industrial applicability. Consequently, scientific research is concentrating on identifying stable, economical and environmentally friendly alternatives to phosphine-based ligands in organometallic catalysis. A particularly promising avenue for exploration involves substituting the phosphorus atom with a chalcogen, notably sulfur (Bhatt et al., 2024; Oswal et al., 2020). This substitution not only enhances the economic viability of the ligands but also ensures their stability in air, a crucial consideration for industrial applications. These novel ligands have the potential to significantly expand the versatility and environmental sustainability of catalytic processes, particularly in enantioselective reactions, where their combination with other atoms, such as nitrogen, is advantageous (Elsby and Baker, 2023; Rajmane and Kumbhar, 2024).

Sulfur-based ligands are part of a broader class of functional (or “actor”) ligands, which exhibit ligand-centered reactivity within the first or second coordination sphere. These ligands can transiently dissociate, rearrange, or undergo chemical transformations during catalysis, influencing both the reactivity of the metal center and their own chemical behavior (Mondal et al., 2022; Shimbayashi and Fujita, 2020).

**FIGURE 1:**
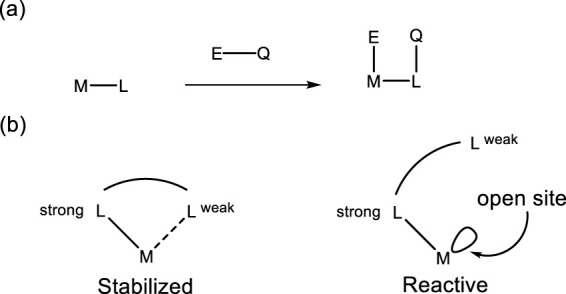
Conventional Metal−Ligand Cooperation mechanism of strongly bonded **(a)** and hemilabile **(b)** ligands.

Much of the design philosophy underlying actor ligands draws inspiration from enzymatic systems such as [FeFe]-, [NiFe]-, and [Fe]-hydrogenases. Within this bio-inspired framework, several strategies are particularly relevant for sulfur-based ligand development. These include cooperative ligands, which directly participate in bond-activation steps and undergo reversible chemical changes alongside the metal center—commonly referred to as metal–ligand cooperation (MLC)—and which may operate in either the inner or outer coordination sphere (Figure 1, box a). Another key class is hemilabile ligands, a subset of cooperative systems featuring one strongly coordinating donor and a second donor that binds weakly and reversibly, thereby providing dynamic access to open coordination sites (Figure 1, box b).

In view of the central role of hydrogenative and dehydrogenative processes in the chemical industry and organometallic catalysis more broadly, this review provides a comprehensive overview of the development of sulfur-based ligand systems in redox-active organometallic complexes. Particular attention is given to catalytic activity, selectivity and stability, emphasizing the crucial impact of electron-rich sulfur ligands on the performance and design of contemporary catalytic processes.

To provide a deeper rationalization and clearer understanding, this review surveys recent literature on sulfur-catalyzed reactions by first organizing catalysts according to the type of (de)hydrogenative process, namely HY, TH and borrowing hydrogen (BH). Within each section, the structural features of the metal complexes of the catalysts are compared, paying particular attention to ligand denticity (mono-, bi-, tri- or tetradentate) and the nature of the coordinating atoms (e.g. thioether, thiolate, thioamide, thiourea and thiophene for sulfur; amine and imine for nitrogen; phosphine and phosphine oxide for phosphorus). Where possible, catalysts are also compared based on the geometry of the complex, e.g. pincer or half-sandwich architectures.

## 2 Hydrogenation

Historically, the reduction of carbon–heteroatom multiple bonds has necessitated the utilization of stoichiometric reducing additives, which are often challenging to manage and have a detrimental effect on the overall greenness of the reaction by influencing pivotal parameters such as atom economy and mass index. Consequently, the development of processes catalyzed by organometallic complexes has been of great importance.

The existing body of literature contains numerous examples of organosulfur ligand-based catalysts that have been demonstrated to be effective in the catalytic HY of carbonyl and carboxylic substrates.

**SCHEME 1:**
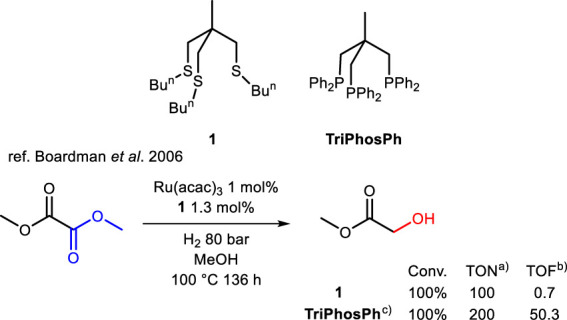
Selective HY of dimethyl oxalate to 2-hydroxyacetate catalyzed by Ru (acac)_3_/TriSulfBu **1** and TriPhosPh systems generated *in situ*. a) TON values are calculated as (moles of products)/(moles of catalyst) or as (moles substrate/moles catalyst) x (conversion). b) TOF (h^-1^) values are calculated from the kinetic constant measured between 20% and 80% conversion. c) Full conversion to ethanediol in the presence of 3 mol% of Zn additive.

In 2006, Boardman et al. published a study that pioneered the attempted replacement of classical phosphorus-based ligands with sulfur-containing alternatives (Boardman et al., 2006). This study developed a self-assembling ruthenium and tripodal SSS ligand **1** in solution, analogous to the one featuring the MeC(CH_2_PPh_2_)_3_ (TriPhosPh) ligand, namely MeC(CH_2_SBun)_3_ (TriSulfBu). The authors successfully employed this system in the selective HY of dimethyl oxalate to methyl glycolate (Scheme 1). It is noteworthy that while the authors did not isolate the complex, the significance of its presence is substantiated by experiments conducted in its absence, which revealed no conversion of dimethyl oxalate. Compared to TriSilfBu, the TriPhosPh ligand in combination with ruthenium and 3 mol% of Zn not only allows to fully convert the dimethyl oxalate to fully reduced ethylene glycol but performs in a faster and more efficient way as can be noticed by TON (turnover number) and TOF (turnover frequency in time^−1^) comparison. Nevertheless, this first attempt demonstrates the feasibility of replacing sulfur with phosphorus and leaves ample room for improvement.

**SCHEME 2:**
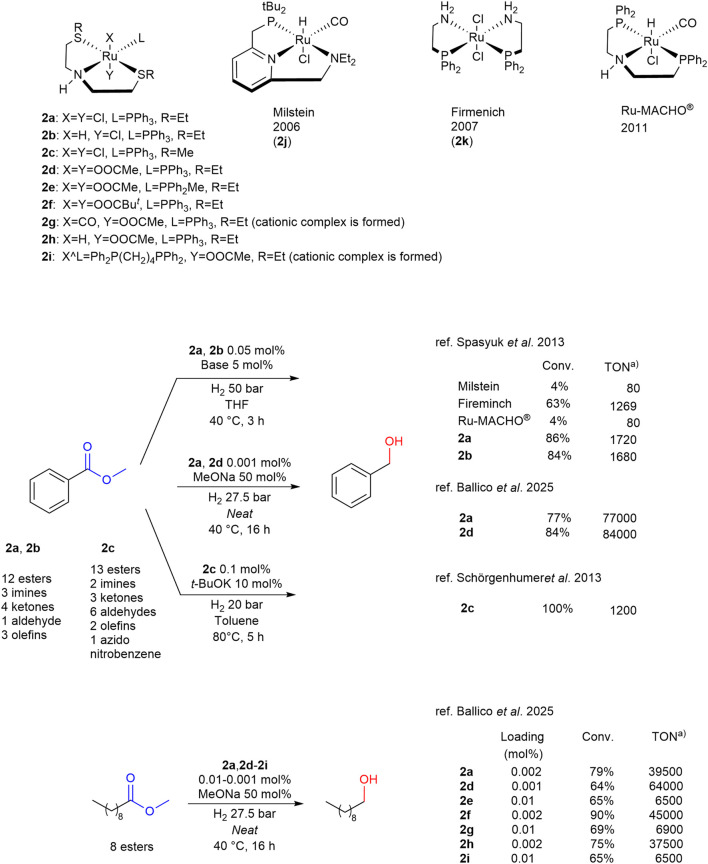
Phosphorus- and sulfur-amino Ru-complexes 2a-k and Ru-MACHO^®^ for C=C, C=N and C=O HY. a) TON values are calculated as (moles of products)/(moles of catalyst) or as (moles substrate/moles catalyst) x (conversion).

The presence of ligands containing mixed chelating centers, which allow for the advantageous properties of multiple heteroatoms to be exploited, has been reported more and more frequently in the literature. An advancement of this approach is found in 2013 in the seminal work of Spasyuk et al. (2013) In this study, the research group synthesized SNS ethylthioether structural analogs (Scheme 2a,b) of the Ru-MACHO^®^ catalyst, which was patented by Takasago in 2011 (Kuriyama et al., 2011). In this study, a comparative analysis was conducted on the catalytic HY of methyl benzoate using a range of reported phosphine-based catalysts, including Milstein’s, Firmenich’s and Ru-MACHO^®^ catalysts, as well as complexes **2a** and **2b**. The results obtained indicated that complexes **2a** and **2b** exhibited higher activity in this transformation, as illustrated in Scheme 2.

Subsequently, complex **2a** was subjected to testing in the context of catalytic HY of esters, ketones, aldehydes, imines and olefins. This investigation led to the expansion of the scope of the reaction and the identification of numerous specific operative conditions. The mechanism by which the complex operates in the reaction is hypothesized by Chen et al. (2016).

In 2018, Schörgenhumer et al. (2018) reported complex **2c**, a structural analogue to complex **2a**, which was utilized with success in the catalytic HY of esters. This process was carried out under much lower hydrogen pressure, higher temperature and catalyst loading and resulted in an expanded reaction scope when compared to the work of Spasyuk et al. 2013 previously reported (Scheme 2). In 2025 Ballico et al. (2025) replaced the chloride anion with carboylates (acetate, pivalate) on the structure of Gusev’s complexes **2a**-**b**, reporting the synthesis of pre-catalysts **2d**-**i** and their application as in the efficient *neat* HY of mostly fatty esters under strongly basic conditions (Scheme 2). As a matter of fact, the most promising complexes bearing PPh_3_ as monodentate ligand **2d** (carboxylate anion) and **2f** (pivalate anion) showed higher productivity with respect to Gusev’s compelxes **2a**-**b**, reaching complete conversions at lower H_2_ pressure (27.5 bar) and Ru-loadings (down to 0.002 mol%). It is worth noticing that the replacement of the PPh_3_ with CO or other phosphine ligands (i.e., PPh_2_Me, Ph_2_P(CH_2_)_4_PPh_2_) resulted detrimental for the catalytic activity and productivity of the system (Scheme 2), as already observed by Gusev and co-workers (Spasyuk et al., 2013).

**SCHEME 3:**
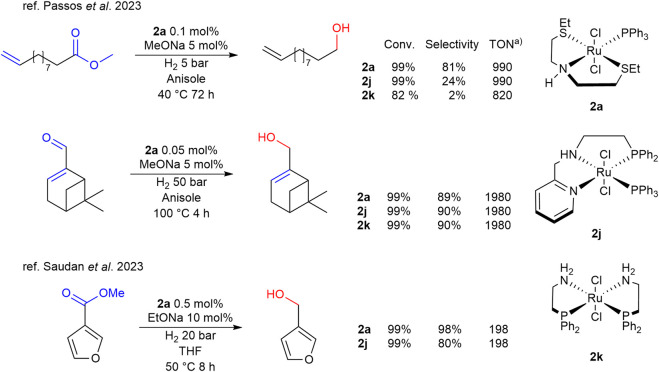
Comparative performances of phosphine- and thioether-based complexes 2a-j in the selective HY of diverse carbonyl moieties a) TON values are calculated as (moles of products)/(moles of catalyst) or as (moles substrate/moles catalyst) x (conversion).

In 2023 the same group (Passos et al., 2023) reported complex **2a** in comparison with main examples of complexes based on phosphine ligands in the HY of unsaturated esters and aldehydes. **2a** was proved to be effective in the reduction of long-chain unsaturated esters, with excellent selectivity and good activity in the case of methyl 10-undecenoate, while in the reduction of *α*,*β*-unsaturated aldehydes it demonstrated an activity and selectivity comparable to the phosphine complexes (Scheme 3). In the same year another study by Saudan and Quintaine (2023) reported the comparison of complex **2a** with various aminophosphine analogues in the HY of heteroaromatic esters. In particular, it was found to be highly efficient and selective in the HY of methyl 3-furanoate to the corresponding primary alcohol (Scheme 3). In this case, after a comparison among the TON values, it is evident how complex **2a** performs usually equally to main used phosphine-based organometallic complexes in such reaction.

**SCHEME 4:**
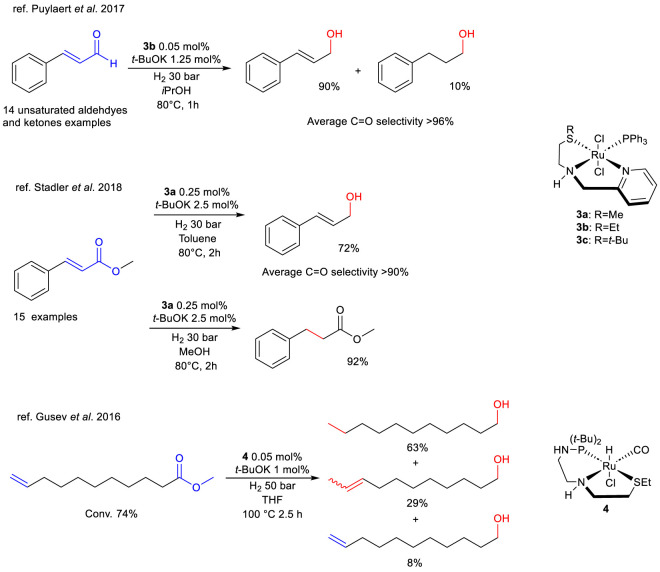
Tunable chemoselective C=C and C=O HY of *α*,*β*-unsaturated aldehydes and esters catalyzed by complexes 3a-c and complex 4.

Another family of complexes employed in catalytic HY comprises structural analogs to Milstein’s catalyst, with the substitution of the residual phosphine in the ligand structure for a thioether group. In 2017, Puylaert et al. (2017) reported the synthesis and application of several AMPY (2-aminomethyl-pyridine)-like NNS thioether ruthenium complexes (**3a**-**c**, Scheme 4) in the catalytic HY of *α*,*β*-unsaturated aldehydes and ketones. In the catalytic HY of *trans*-cinnamaldehyde, complexes have been compared, and complex **3b** has been identified as the most active. This complex achieved 100% conversion in 1 h, with high selectivity for the C=O bond reduction (Scheme 4). Complex **3b** has been subjected to testing in the aforementioned conditions in the catalytic HY of many unsaturated aldehydes and ketones, with a generally high conversion and selectivity for the C=O bond >96%.

In 2018, the same research group (Stadler et al., 2018) reported that the reactivity of complexes **3a** and **3c** can be modulated. Interestingly, the study demonstrated that the selectivity towards the C=C and the C=O is readily adjustable by modifying the operational conditions, such as the solvent (Scheme 4). Testing of complex **3a** in the catalytic HY of many *α*,*β*-unsaturated and simple esters in toluene has shown a general high conversion and selectivity for the C=O bond of over 90%. In methanol the formation of the alkene HY is selective to achieve complete conversion and very high selectivity.

Another study that reported not only efficiency but also selectivity of sulfur based Ru catalyst has been published in 2016 by Gusev (Gusev, 2016), which presents a PNS aminophosphino thioether ligand in Ru-complex **4**. It was tested in the HY of methyl 10-undecenoate and resulted in good overall activity (74% conv.) with moderate selectivity towards the–COOR moiety (Scheme 4). Interestingly, a significant amount of C=C isomerized alcohol products (9-undecenols) was detected in the reaction mixture, disclosing an unprecedented reactivity for this type of thioether-based complexes.

**SCHEME 5:**
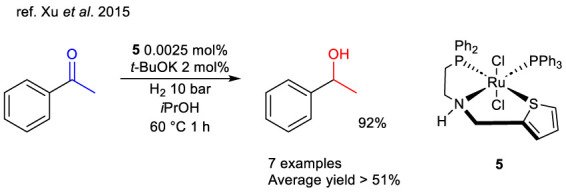
HY of aromatic ketones catalyzed by complex 6.

In 2015 Xu and Langer (2015), described the effect of the weaker coordination conferred by the thiophene donor group on the catalytic activity of a Ru-based complex in the HY of aldehydes and ketones. Excellent conversions of acetophenone under mild conditions and in a short time were obtained with complex **5** (Scheme 5), although the versatility of the complex and of the operating conditions was not proven, with generally reduced activity except for aliphatic ketones.

**SCHEME 6:**
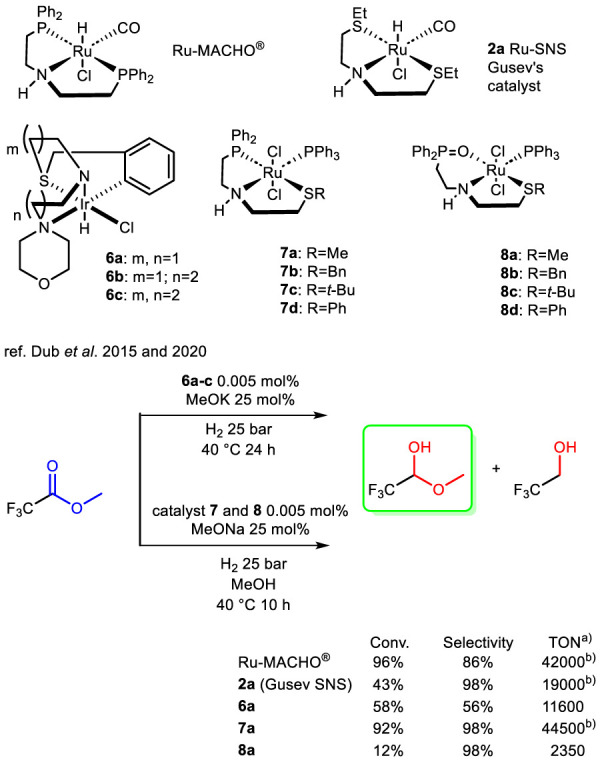
Semi HY of methyl trifluoroacetate to its methyl hemiacetal catalyzed by complexes 2a, 6–8. a) TON values are calculated as (moles of products)/(moles of catalyst) or as (moles substrate/moles catalyst) x (conversion) b) Calculated using 0.002 mol% of complex after 24 h.

In the search of alternative to the ruthenium catalyst in 2015, Dub et al. reported 15 examples of well-defined Ru and Ir NNS-like complexes. The catalysts were compared in the HY of methyl trifluoroacetate to trifluoro acetaldehyde methyl hemiacetal (Dub et al., 2015). Aim of this study was to investigate air- and moisture-stable complexes and interestingly among all the catalysts, iridium-based complexes **6a-c** demonstrated the highest activity in this reaction (Scheme 6), with an efficiency that was only slightly lower than that of the most effective phosphorus-based bifunctional catalysts. The catalyst reached a turnover number >10,000 under mild conditions (40 °C, 25 bar H_2_) in basic methanol.

In 2020, the same group published a in a study in which many ruthenium SNP(O)_x_ (x = 0,1) complexes were reported as a more product selective alternative to the most commonly used Ru-MACHO^®^ (Kuriyama et al., 2012) and Ru-Gusev (SNS) complexes **2a** (Spasyuk et al., 2013) in hydrogenative processes, *i*.*e*. the HY of *α*-fluorinated esters (Dub et al., 2020). Complexes **7a-d** and **8a-d** were tested in the same reaction reported for the iridium catalyst and compared with the main references mentioned above (Scheme 6). Complexes **7a-d** were generally as active as Ru-MACHO^®^ but more selective for the hemiacetal form, while complexes **8a-d** were generally less active than **2a**. The lower activity of complex line **8** could probably be attributed to a poisoning effect of the phosphine oxide group compared to phosphine. In particular, the best catalyst resulted in complex **7a**, and its versatility in HY protocols was demonstrated with generally excellent activity and selectivity (Scheme 6) compared to the main relevant catalysts in the literature.

Despite numerous examples of sulfur-based pincer ligands used to construct organometallic complexes that have been successfully employed in catalytic HY reactions, the same cannot be said for half-sandwich complexes.

**SCHEME 7:**
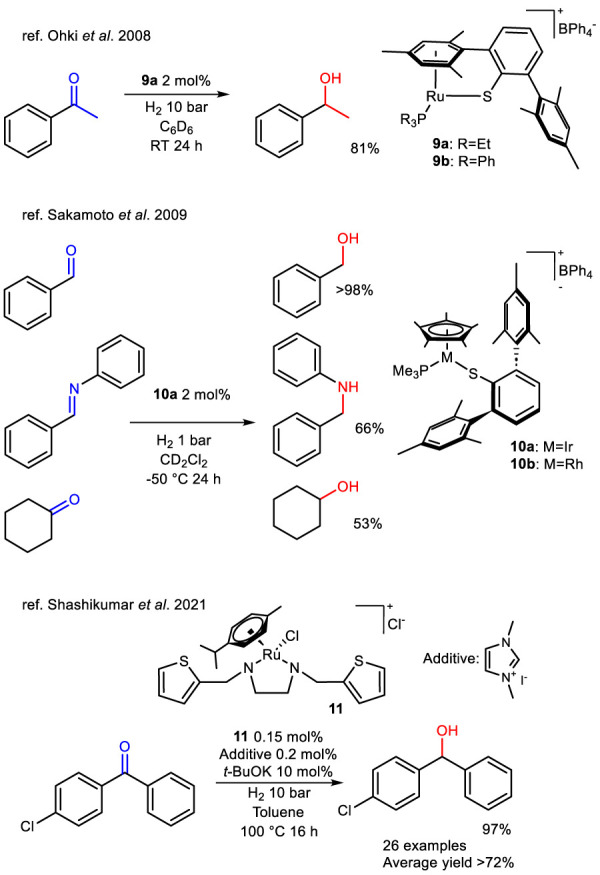
HY of ketones, benzaldehyde and (*E*)-*N*,1-diphenylmethanimine catalyzed by half-sandwich thiolate Ru, Ir and complexes 9, 10 and thiophene-based Ru-complex 11.

Studying metal–thiolate complexes in metalloenzymes, where both the metal center and sulfur atoms actively participate in reactions, provides valuable insights for designing new catalysts. A notable development in this field was the creation of a coordinatively unsaturated metal center with a terminal, bulky thiolate ligand. In 2008, Ohki et al. reported a half-sandwich ruthenium complex (complex **9a**) featuring this design (Ohki et al., 2008b). Due to the electrophilic nature of the metal and the nucleophilic character of sulfur, it was hypothesized that complex bearing thiolate ligands exploits metal–sulfur bonds in substrate activation, such as the heterolysis of H_2_. Notably, complex **9a** effectively catalyzed the HY of acetophenone (Scheme 7), but exposure more than 200 eq of substrate caused its degradation into catalytically inactive derivatives. In their study, the authors proposed a mechanism in which H_2_ undergoes heterolysis and coordinates at the vacant ruthenium site, generating a Ru–H/S–H intermediate. In agreement with the mechanism established for Noyori-type ruthenium catalysts, this species may transfer both a hydride and a proton to acetophenone in a concerted manner (Sandoval et al., 2006).

An evolution of the system just described was reported in 2009 by Sakamoto et al. (2009) Half-sandwich thiolato complexes of rhodium and iridium were synthesized and tested in the HY of C=O and C=N bonds (Scheme 7). Complex **10b** showed low activity even when the reaction time was doubled, while complex **10a** was moderately efficient under relatively mild reaction conditions. The main limitation of this protocol is the need to operate at very low temperatures (−50 °C for **10a** and −20 °C for **10b**), as the active hydride species were found to be unstable even at room temperature. Another example of this system was reported in 2021 by Shashikumar et al. (2021) who described the synthesis of a phosphine-free ruthenium half-sandwich complex **11** with an ethylenediamine ligand bearing two weakly coordinating thiophene arms. This complex was obtained with the aim to obtain a cost-effective catalyst that is air stable, the catalyst was successfully tested in the HY of 4-chlorobenzophenone, where generally the addition of an N-heterocyclic carbene ligand was found to be essential. The complex has been compared with non-heteroaromatic analogues and it has been noted that the thiophene presence is essential for the activity of the system. NHC ligand as an additive were shown to enhance the efficiency of molecular HY. The reaction scope was subsequently extended, demonstrating the versatility of the system in the HY of various ketone, aldehyde and imine substrates (Scheme 7) nevertheless the excellent activity was maintained only with benzophenone-like substructures. Interestingly, complexes **9a**-**b** and **10a**-**b** showed high catalytic activity in the absence in a base-free medium, whereas **11** needed the addition of a strong base for its activation.

**SCHEME 8:**
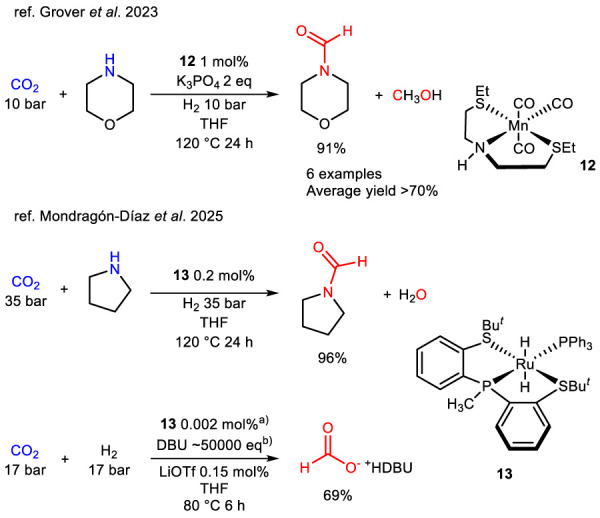
Indirect and direct HY of CO_2_ assisted by cyclic amines catalyzed by 12, 13 and assisted by DBU catalyzed by 13, respectively.

Another interesting approach to investigate catalytic activity of metal-sulfur catalysts is the indirect HY of CO_2_. When assisted by an amine, this process leads to the formation of a formamide derivative. In 2023, Grover et al. (Grover et al., 2023) reported complex **12**, an SNS pincer Mn complex active in the indirect HY of CO_2_ to *N*-formyl morpholine and methanol under relatively mild conditions. The reaction scope was subsequently extended by subjecting various cyclic and primary amines to the optimized conditions, with good results (Scheme 8).

Another example of this type of reaction is represented by the ruthenium-based SPS complex **13**, reported by Mondragón-Díaz et al. (2025). This complex demonstrated activity in both the indirect HY of CO_2_ with pyrrolidine to afford *N*-formyl pyrrolidine and in its direct HY. When the reaction was performed with a large excess of 1,8-diazabicyclo [5.4.0]undec-7-ene (DBU) as an additive it yielded formic acid salt. This activity confirms the remarkable versatility of **13** (Scheme 8).

**SCHEME 9:**
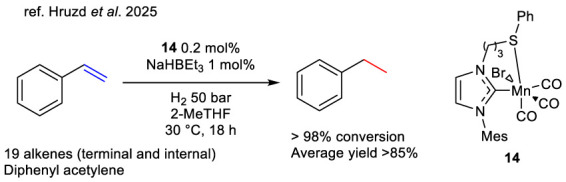
HY of C–C multiple bonds catalyzed by 14.

Very interesting is the study presented in a recent work of Hruzd et al. (2025) that approaches the preparation, characterization and application of phosphine free SNS metal complex catalysts for HY at room temperature. A manganese SC thioether complex **14** was described, among the very few sulfur-based complexes successfully employed in the catalytic HY of olefins. The study demonstrates that the use of NHC–thioether ligands enables the selective HY of alkenes at room temperature with catalytic loadings as low as 0.2 mol%, under milder conditions than with P–NHC–P or bis-NHC Mn complexes (Scheme 9). The scope of this protocol was extended to 19 alkenes either terminal and internal and 1 alkyne. Yields were extremely high and the catalyst was exploited in 0.1–1 mol%.

## 3 Transfer hydrogenation

### 3.1 Alcohols as H_2_-donors

Transfer hydrogenation (TH) is an evolution of the more conventional catalytic HY process in which the use of a hydrogen donor (typically acting as a solvent) rather than pressurized molecular hydrogen allows for a significant simplification of the required reaction equipment while reducing the inherent health risks associated with hydrogen handling. As this approach is inherently user-friendly, its further development is being pursued with a focus on environmental sustainability, particularly in terms of waste reduction. This is being achieved by designing highly efficient systems that minimize the amount of catalyst or additives required, and by developing processes that can operate in environmentally friendly solvents such as water or glycerol.

Numerous examples of organosulfur ligand-based catalysts active under TH conditions have been reported in the literature in recent years, differing both in their metallic core and in the nature of the functional group carrying the sulfur atom. In this review, we analyze the performance of these catalysts in the TH of acetophenone. This was chosen as a reference system for evaluation and comparison based on turnover number (TON) and turnover frequency (TOF) measurements. It should be noted, however, that these catalysts have also been reported to be versatile and can be used for the TH of various ketones, aldehydes, nitriles and nitro compounds.

**FIGURE 2:**
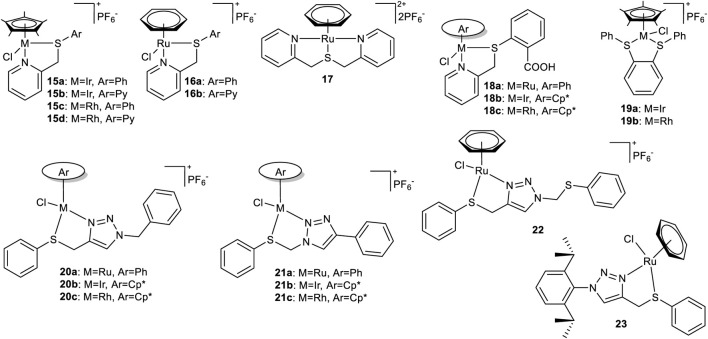
Thioether-based half-sandwich complexes bearing heterocycles.

The majority of these complexes are constituted by thioether ligands of either the SN or SNN type. A further classification of these complexes is possible according to the nature of the nitrogen atoms, thus distinguishing between heterocyclic, iminic and aminic forms (Figure 2).

Prakash et al. report in their studies the synthesis of pyridine-based thioether ligands and their development into iridium, rhodium, and ruthenium complexes. Their structures are reported in Figure 2 and their activity over TH of acetophenone is reported in Table 1. The research group compares these ligands with their selenium-based analogs. The iridium-based complexes **15a-b** and the rhodium-based complexes **15c-d** are reported in the 2012 study (Prakash et al., 2012). In this article, the authors undertake a comparative analysis of the catalytic activities exhibited by these two metals within the specified system. Additionally, they explore the impact of various sulfur-bound substituents, specifically phenyl and pyridine, on the catalytic activity of the complexes. The study concludes that the rhodium-based complexes exhibit higher activity, achieving greater conversion in a shorter time compared to its iridium counterpart. Furthermore, the investigation reveals that in presence of thioether ligands (complex **15a** vs. **15b** or **15c** vs. **15d** Table 1, entries 1–4), *S*-phenyl substituent has an improved efficacy if compared to S-pyridine as demonstrated from the evaluation of TON values of acetophenone TH. The complex **15c** was identified as the most efficient organosulfur catalyst. In the 2013 study (Prakash et al., 2013), the previously described ligands were also tested with ruthenium in complexes **16a-b**, alongside the introduction of complex **17**, a symmetric tridentate analog. The study reports that the catalytic activity of ruthenium in the bidentate complexes is fully comparable to that of the previously described rhodium-based analogs. However, the tridentate ligand had a detrimental effect on metal efficiency, requiring a tenfold increase in the catalyst loading to achieve the same conversion level as complex **16a** (Table 1, Entry 6). An evolution of this system is described in their 2014 study (Prakash et al., 2014a), in which the research group reports on complexes **18a-c**, differing in their metal center. The introduction of a carboxyl group on the aromatic substituent enabled the TH of acetophenone to be conducted in water using stoichiometric amounts of glycerol as a hydrogen donor. This process was found to be a greener alternative compared to other commonly used alcohols or additives. In this system, the rhodium-based complex **18c** proved to be significantly more efficient than its ruthenium-based counterpart **18a** (Table 1, entries 7–8). It is important to note that this process necessitated a substantial increase in catalyst and KOH loading, as well as higher temperatures and extended reaction times, which had a negative impact on the TON and TOF values. However, this stands as a rare example of organosulfur ligand-based catalysts being successfully employed with glycerol as a hydrogen donor. Moreover, to the best of our knowledge, this is one of the only two reported cases where the reaction is performed in water, the most environmentally friendly solvent, using only stoichiometric amounts of glycerol, thereby drastically reducing reaction waste. Finally, although structurally different from the previously described analogs, it is important to mention non-heteroaromatic complexes **19a-b**, reported by the same research group in the same year (Prakash et al., 2014b). These complexes represent an example of catalysts active which is active in the TH of acetophenone, with glycerol functioning as both the solvent and hydrogen donor. However, it should be noted that, as in the previous case, more drastic conditions were required in this instance when compared to the protocols conducted in 2-propanol. Saleem et al. report in their studies the synthesis of easily feasible click-chemistry triazole-based thioether ligands and their development into iridium, rhodium, and ruthenium complexes **20–23**. As reported in Table 1 the comparison of the complex **20a**, in which chelation occurs at N-3, and complex **21a**, in which chelation occurs at N-2 resulted in superior activity in TH of acetophenone of the latter due to the enhanced chelating and stabilizing ability of the N-2 nitrogen (Table 1, entries 10 and 13) (Saleem et al., 2013). In 2014 the same research group (Saleem et al., 2014), the authors sought to expand the applicability of the previously described ligands by developing analogous iridium-based complexes **20b** and **21b**, as well as rhodium-based complexes **20c** and **21c**. The complex **21c** showed exceptional efficiency in the TH of acetophenone with a tenfold reduction in the required catalyst loading compared to iridium and ruthenium analogues (Table 1, entries 10–15), coherently with the iridium complexes there is no strong evidence of a difference between the activity of the N-2 and the N-3 chelating Rh complexes.

**TABLE 1:** Comparison of catalytic activities of complexes **15–23** in the TH of acetophenone.

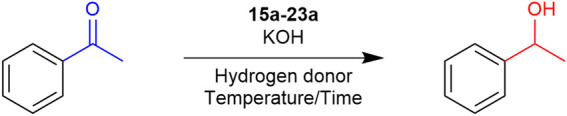							
En^a^	Cat	Cat. (Mol%)	KOH (eq.)	Temp/Time	Conv. (%)	TON^b^	Ref.
1	**15a**	0.01	0.4	80 °C/5 h	88	8800	Prakash et al. (2012)
2	**15b**	0.01	0.4	80 °C/5 h	80	8000	
3	**15c**	0.01	0.4	80 °C/3 h	95	9500	
4	**15d**	0.01	0.4	80 °C/3 h	85	8500	
5	**16a**	0.01	0.4	80 °C/3 h	95	9500	Prakash et al. (2013)
6	**17**	0.1	0.4	80 °C/3 h	94	940	Prakash et al. (2013)
7	**18a**	1	1	110 °C/20 h^c^	90	90	Prakash et al. (2014a)
8	**18c**	1	1	110 °C/20 h^c^	98	98	
9	**19b**	1	3	120 °C/24h^d^	60	60	Prakash et al. (2014b)
10	**20a**	0.1	0.4	80 °C/3 h	93	930	Saleem et al. (2013)
11	**20b**	0.01	0.4	80 °C/3 h	85	8500	Saleem et al. (2014)
12	**20c**	0.001	0.4	80 °C/3 h	68	68000	
13	**21a**	0.01	0.4	80 °C/3 h	97	9700	Saleem et al. (2013)
14	**21b**	0.01	1	80 °C/3 h	85	8500	Saleem et al. (2014)
15	**21c**	0.001	1	80 °C/3 h	70	70000	
16	**22**	0.01	1	80 °C/3 h	87	8700	Saleem et al. (2015)
17	**22**	0.5	1	80 °C/3h^d^	82	164	
18	**23**	0.4	1	110 °C/6 h	70	175	Kumar et al. (2016)

^a^ Reactions are performed in *i*PrOH, as the hydrogen donor, except for entries 7, 8, 9, and 17.

^b^ TON, values are calculated as (moles of products)/(moles of catalyst) or as (moles substrate/moles catalyst) x (conversion)

^c^ Hydrogen donor is Gly (1 eq).

^d^ The hydrogen donor is Gly (solvent).

In the 2015 study (Saleem et al., 2015), the authors introduce complex **22**, a ruthenium-based evolution of complex **20a**, in which the ligand structure is expanded by incorporating a second sulfur atom. This modification increases the versatility of the complex class, enabling the TH of acetophenone using glycerol as both a solvent and a hydrogen source (see Table 1, entries 16–17). However, it was observed that a significantly higher catalyst loading was required in the presence of glycerol. Finally in 2016, Kumar et al. reported the ruthenium-based complex **23** containing triazole-based thioether ligands, Kumar et al. (2016) tested in the TH of acetophenone using water as solvent and glycerol as hydrogen source in stoichiometric amount and showing lower conversion under harsher reaction conditions with respect to its thio-based congeners (Table 1, entry 18).

**SCHEME 10:**
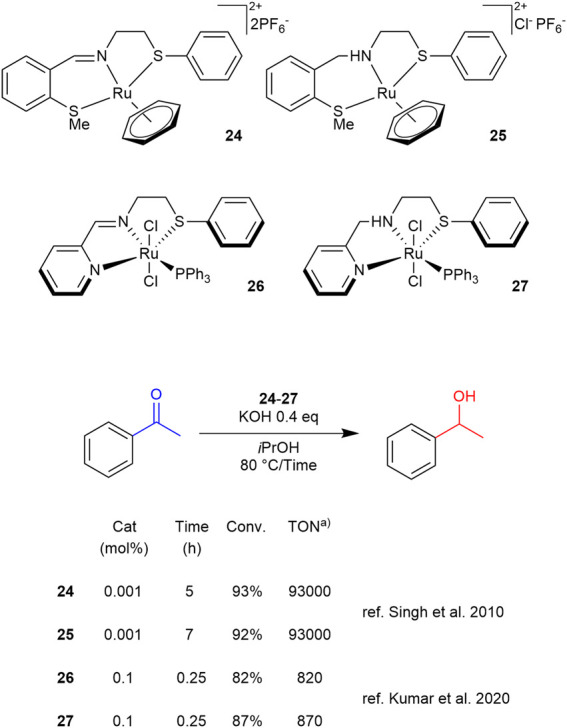
TH of acetophenone catalyzed by 24–27. a) TON values are calculated as (moles of products)/(moles of catalyst) or as (moles substrate/moles catalyst) x (conversion).

**SCHEME 11:**
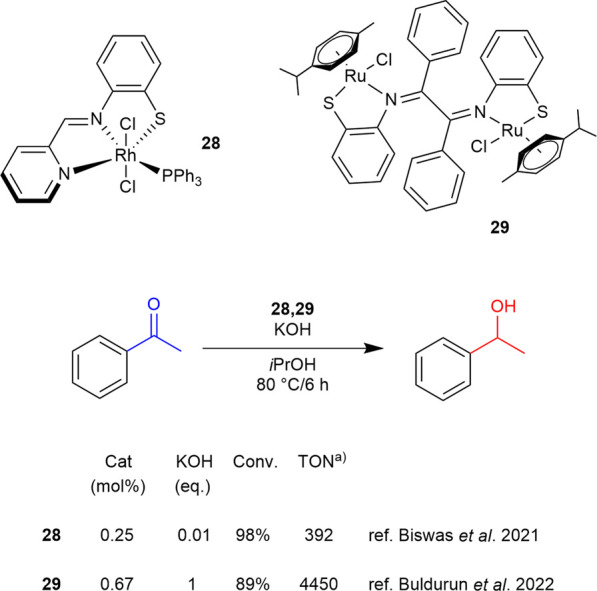
TY of acetophenone catalyzed by thiolato complexes 28 and 29 a) TON values are calculated as (moles of products)/(moles of catalyst) or as (moles substrate/moles catalyst) x (conversion).

**SCHEME 12:**
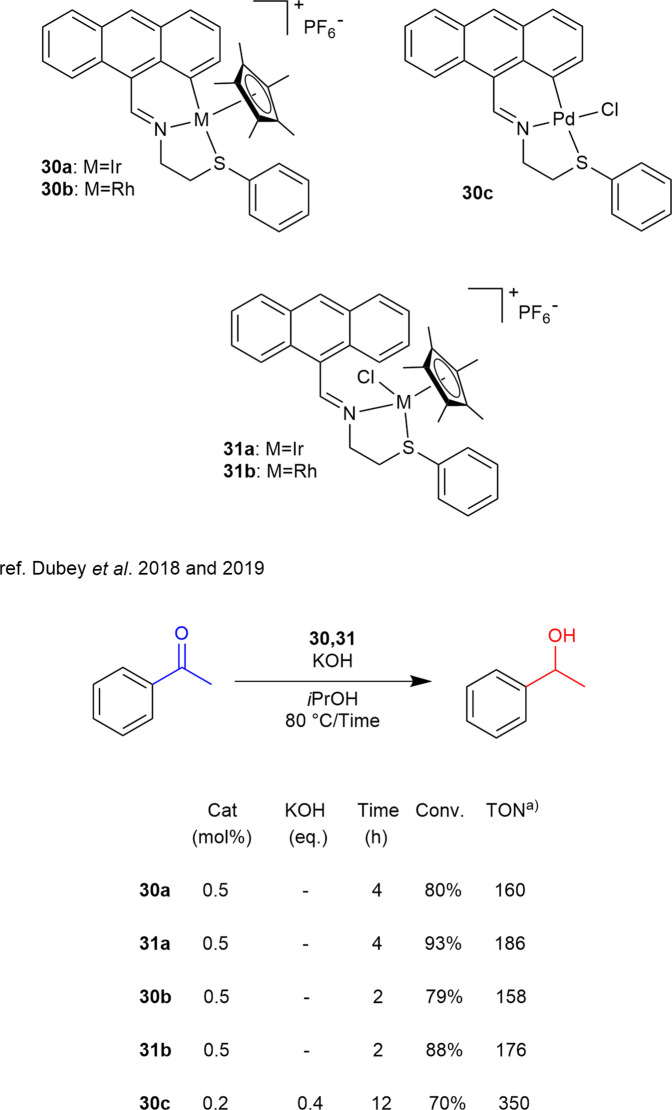
TH of acetophenone catalyzed by catalysts 30a,b and 31a,b. a) TON values are calculated as (moles of products)/(moles of catalyst) or as (moles substrate/moles catalyst) x (conversion).

In the series of complexes synthesized and tested for TH with thioether ligands, an interesting comparison emerges between SN and SNN ligand complexes, depending on whether the chelating nitrogen atom is present in its imine or amine form. As shown in Schemes 10–12, three pairs of complexes (**24**–**27** and **26**–**27**) illustrate this amino/imino variation. Interestingly, not only ruthenium but also iridium and rhodium complexes are represented.

A pioneering example of this class is represented by the SNS ruthenium-based complexes **24** and **25**, which feature an imine and an amine moiety, respectively, as reported by Singh and Singh (2010). These complexes have proven to be highly efficient in the TH of acetophenone, requiring a very low catalyst loading and achieving a TON exceeding 90000, establishing **24** and **25** as the most active complexes in this category. Curiously, complex **24** exhibited higher activity than its amine counterpart **25**, reaching the same conversion in a shorter reaction time (Scheme 10).

When compared with the tridentate NNS ruthenium-based complexes **26** and **27** (Kumar et al., 2020), the presence of an additional chelating nitrogen in their framework allows for a substantial reduction in reaction times for the TH of acetophenone, at least eight times shorter than those reported for other complexes in this type of reaction (Scheme 10). In this context, in contrast to the observations made for complexes **24** and **25**, complex **27**, which features a chelating nitrogen of amine nature, proved to be slightly more efficient than its imine counterpart, hinting that the latter may be reduced to the former moiety under the TH conditions after a brief induction period.

A structural simplification of the previously described NNS system was achieved by replacing the thioether moiety with a thiolate group. An example is reported in 2021 by Biswas et al. for a rhodium-based complex **28**, Biswas et al. (2021) it represents the first attempt in this direction. While its activity in TH was moderate, it enabled a significant reduction in the amount of base required for this transformation with excellent conversion at 80 °C with 0.01 eq of KOH (Scheme 11). In 2022 Buldurun et al. (2022) described the evolution of this thiolate-based system, culminating in the bimetallic symmetric ruthenium complex **29**, which exhibited good activity in TH while requiring a lower catalyst loading compared to the other reported examples (Scheme 11). Based on these results and in accordance with their TON values, thiolate-based complexes exhibit diminished efficiency in comparison to their thioether counterparts. In fact, the latter have undergone considerably more extensive development over the years.

In the domain of complexes that explored the comparison amine/imine ligands, a variation on this theme is represented by complexes **30a**-**c** and **31a**-**b**, described by Dubey et al. (2018) and Dubey et al. (2019) respectively, which are tri- and bidentate, and feature an anthracene moiety. The complexes refer to iridium and rhodium while complex **30** also palladium. Despite the lower TON values of these complexes in the TH of acetophenone (Scheme 12), to the best of our knowledge they represent, with the exception of the Pd-based complex **30c**, the only example of base-free TH was achieved using organosulfur-ligand-based complexes. Notably, the bidentate complexes **31a–b** demonstrated higher efficiency than their tridentate counterparts **30a–b**. Moreover, the Rh-containing complexes (**30b** and **31b**) outperformed their iridium analogues, resulting in higher conversion.

Compared to the thioether- and thiolate-based organosulfur-ligand complexes discussed so far, the development of systems in which the chelating sulfur is incorporated within different functional groups applicable to TH reactions has been less successful, with only a few reported examples that are generally less efficient than the previously discussed systems.

**FIGURE 3:**
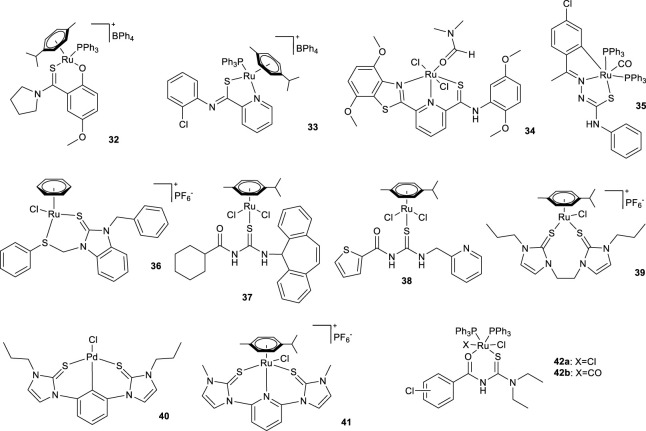
Thioamide and thiourea coordinating complexes.

Two notable classes in this category are those in which the chelating sulfur is contained within a thioamide or thiourea (Figure 3) functional group. Their reactivity in the TH of acetophenone is compared in Table 2.

**TABLE 2:** TH of acetophenone and nitrobenzene catalyzed by complexes **32**–**42**.

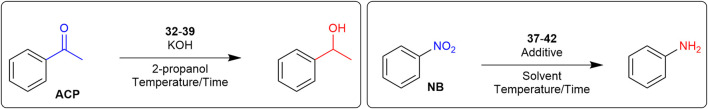							
En	Cat	Cat. (Mol%)	Additive eq, solvent^a^	Temp./Time	Conv. (%)	TON^c^	Ref.
1	**32**	0.1	ACP, KOH 0.005 eq, *i*PrOH	80 °C/8 h	97	970	Pandiarajan and Ramesh (2013)
2	**33**	0.1	ACP, KOH, 0.4 eq, *i*PrOH	82 °C/2 h	97	970	Kanchanadevi et al. (2016)
3	**34**	0.2	ACP, KOH, 0.02 eq, *i*PrOH	82 °C/48 h	97	485	Lorraine et al. (2020)
4	**35**	0.2	ACP, KOH, 0.4 eq, *i*PrOH	80°/2 h	99	497	Pandiarajan and Ramesh (2011)
5	**36**	0.1	ACP, KOH, 0.4 eq, *i*PrOH	80 °C/3 h	94	940	Sharma et al. (2014)
6	**37**	0.5	ACP, NaOH, 1 eq *i*PrOH	82 °C/18 h	99	198	Rohini et al. (2018)
7	**37**	0.5	NP, NaOH 1eq, *i*PrOH	82 °C/18 h	99	198	
8	**38**	0.1	ACP, NaOH, 1 eq, *i*PrOH	82 °C/14 h	99	990	Sathishkumar et al. (2018)
9	**38**	0.1	NP, NaOH, 1 eq, *i*PrOH	82 °C/16 h	46	460	
10	**39**	0.5	ACP, KOH, 0.1 eq, *i*PrOH^b^	82 °C/6 h	68	137	Ywaya et al. (2024)
11	**40**	1	NP, NaBH_4_ 4 eq, EtOH	r.t./2 h	90	90	Jia et al. (2019)
12	**41**	0.25	NP, NH_3_·BH_3_ 4.3 eq, H_2_O:MeCN 40:1^b^	80 °C/1 h	99	396	Jia et al. (2020)
3	**42a-b**	1	NP, NaBH_4_ 4eq, EtOH	50 °C/0.5 h	99	99	Uysal et al. (2023)

^a^ ACP = acetophenone; NB = nitrobenzene.

^b^ 7 mol% hexadecyl trimethyl ammonium bromide (CTAB).

^c^ TON, values are calculated as conversion/(catalyst loading percentage) or as (substrate/catalyst ratio) * conversion.

Considering complexes with a thioamide-like structure reveals that complexes **32** (reported by Pandiarajan and Ramesh (2013), **33** reported by Kanchanadevi et al. (2016) and **34** (Lorraine et al., 2020) have demonstrated moderate activity in the TH of acetophenone (Table 2, entries 1-3, respectively). It is worth noting the observation that complex **32** allowed the progression of the reaction with extremely low amounts of KOH, thereby concomitantly effecting a reduction in the waste material generated during the process.

In consideration of the complexes that possess a thiourea-like structure, specifically complexes **35** (Pandiarajan and Ramesh, 2011) and **36** (Sharma et al., 2014), it is evident that they have once again exhibited moderate activity in the TH of acetophenone (Table 2, entries 4 and 5, respectively). On the other hand, although relatively high catalyst loading is required, the reaction times remain relatively short.

Despite the modest activity of the thiourea-like structured complexes in the TH of carbonyl substrates, they have proven to be more versatile than the other examples reported, showing activity also in the TH of nitro compounds and, as previously discussed, the studies demonstrate the effectiveness of these complexes on structurally diverse substrates.

Complexes **37** (Rohini et al., 2018) and **38** (Sathishkumar et al., 2018), under conditions fully comparable to those employed for the TH of carbonyl compounds, were able to reduce nitrobenzene to aniline. Complex **37** demonstrated comparable activity for both reactions with high selectivity, whereas **38** exhibited higher productivity with a significant higher activity and selectivity for acetophenone reduction (Table 2, entries 7–9). Interestingly, when a nitroaryl aldehyde or ketone were subjected to TH catalyzed by **37** or **38**, only nitro reduction to amine was observed, leaving the carbonyl functionality unreacted.

Bis(imidazole-2-thione) ligands gained attention as soft sulfur donors to produce half-sandwich complexes with characteristic piano-stool architecture. As reported in Table 2, entry 10, complex **39** showed good activity in the catalysis of TH of acetophenone and its performance is influenced by alkyl spacer length between the two imidazole-2-thione and the steric bulk of the *N*-substituents (Ywaya et al., 2024). Another interesting example is reported by Jia et al. referring to a tridentate thiourea-based complexes that feature an SXS (X = C, N) coordination motif (Jia et al., 2019). In 2019, the palladium-based complex **40** was described as active in the TH of many aromatic and aliphatic nitro compounds at room temperature and within a short reaction time (Table 2, entry 11). Ethanol was used as the solvent and in combination with NaBH_4_ as the hydrogen source (proton from ethanol and hydride from NaBH_4_). In 2020, the ruthenium-based complex **41** (Jia et al., 2020) was reported to be active in the TH of many aromatic and aliphatic nitro compounds. Despite the elevated level of complexity in the reaction conditions when compared to their previous studies, the authors effectively adapted the protocol to operate within aqueous media by strategically incorporating catalytic amounts of a phase-transfer catalyst (Table 2, entry 12).

The most recent example of thiourea-based complexes successfully employed in the TH of nitro compounds is described in the 2023 study by Uysal et al. The study involves OS-type ruthenium complexes **42a-b** (Uysal et al., 2023), which are active in the reduction of various substituted nitrobenzenes at low temperatures.

Generally, the nitro group reduction mediated by complexes **39**–**41**, **42a**-**b** exploits the combined action of the system ethanol/NaBH_4_ as the hydrogen source.

**SCHEME 13:**
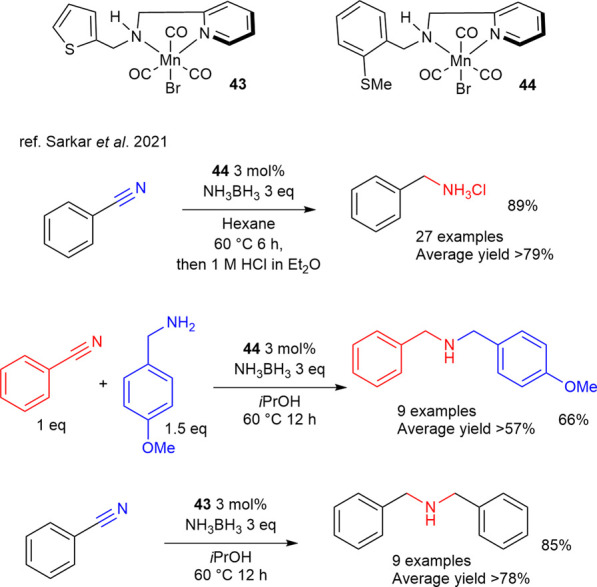
Double TH catalyzed by manganese SNN complexes 43 and 44.

A distinctive application of TH is reported in the 2021 work by Sarkar et al. (2021) which describes the phosphine-free Mn SNN complexes **43** and **44**, bearing a thiophene and a thioether, respectively, and active in a double TH process over nitrile functional group mediated by ammonia-borane (Scheme 13).

In this work the distinct reactivity of complexes **43** and **44** has been proved toward various aromatic and aliphatic nitriles. Specifically in presence of ammonia-borane, complex **44** was reported to catalyze the full TH reduction of nitriles to primary amines selectively in hexane as solvent with efficiency, while in the presence of an excess of a differently substituted primary amine the immine intermediate is prone to react and to provide the addiction product. The imine formed from the first TH of the nitrile undergoes condensation with the primary amine in excess concurrently with ammonia elimination to give a *N*-substitute immine which then is reduced to give non-symmetric secondary amines. Complex **44**, instead, is described in the synthesis of symmetrical secondary amines by generating both imine and primary amines in the reaction mixture in presence of 2-propanol as solvent, leading to a reactivity similar to the one described above.

### 3.2 Silane- and borane-promoted transfer hydrogenation

**SCHEME 14:**
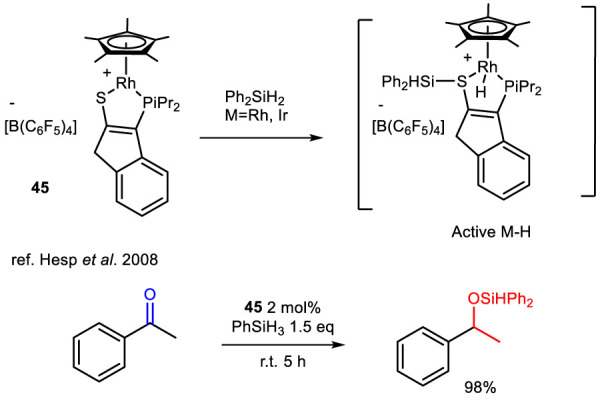
Generation of the catalytically active M–H species from complex 45 and hydrosilylation of acetophenone.

The use of transition-metal complexes featuring a metal–sulfur (M–S) bond has been extensively studied for their ability to promote cooperative metal–ligand Si–H bond cleavage. In these systems, the metal–sulfur bond combines the Lewis acidity of the metal center with the Lewis basicity of the adjacent sulfur atom. This cooperative interaction enables the heterolytic cleavage of Si–H and B–H bonds, generating a metal hydride and a sulfur-stabilized silyl (Si^+^) or boryl (B^+^) cation (Omann et al., 2017). In 2008, this strategy was successfully employed with a set of catalysts tested for their ability to act as donors of silyl cations and hydrides during the hydrosilylation of acetophenone to produce phenylethanol silyl ether (Hesp et al., 2008). Among this group of metal thiolato complexes (Scheme 14), catalyst **45** was shown to quantitatively reduce an acetophenone derivative in the presence of an equimolar amount of organosilane during the dehydrogenative coupling of carbonyl compounds. The catalytically active species was identified as a metal hydride intermediate (active M–H).

**SCHEME 15:**
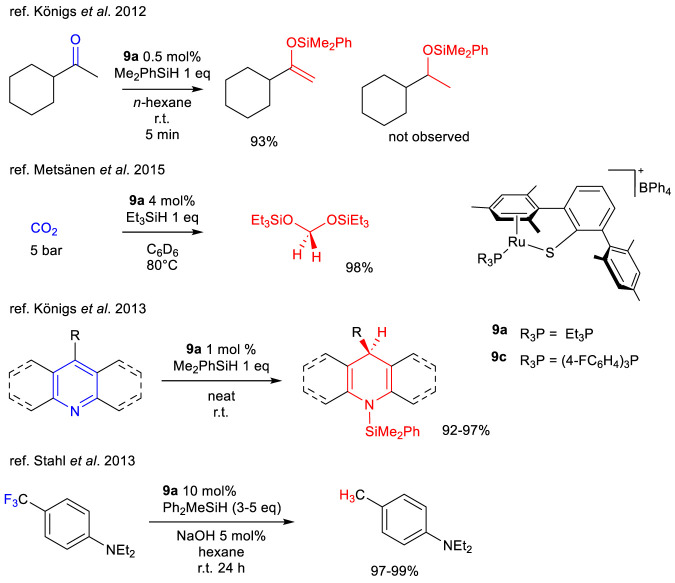
Dehydrogenative silylation of ketones, hydrosylilation of CO_2_ and pyridines and hydrodefluorination of trifluoroarenes with silanes catalyzed by complex 9a.

In this field great contribution have been made by Oestreich, et al that exploited a thiolate-tethered ruthenium arene complex to perform various transformations: dehydrogenative couplings, chemoselective reductions and hydrodefluorination reactions. More specifically the catalyst **9a** has been proved to efficiently catalyze dehydrogenative silylation of enolizable carbonyl compounds to obtain silyl enol under neutral conditions (Königs et al., 2012), and redox processes such as 1,4 hydrosilylation of pyridines to dihydropiridine (Königs et al., 2013), reduction of CO_2_ to the diethyl acetal (Metsänen and Oestreich, 2015) and hydrodefluorination of ortho para substituted anilines (Stahl et al., 2013) (Scheme 15). Interestingly, the Ru–S catalyst **9a** primarily acts as a base rather than a hydride donor—a seemingly contradictory behavior that is, however, well documented in several dehydrogenative bond-forming reactions (Thankachan et al., 2022). Hydride transfer from this catalyst has only been observed when no acidic hydrogen atoms are present.

**SCHEME 16:**
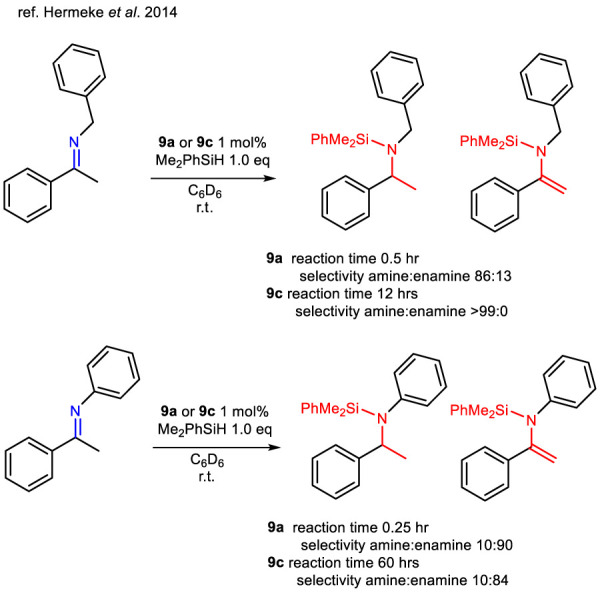
Selectivity in the hydrosilylation of ketimines with Me_2_PhSiH catalyzed by complexes 9a,c.

In general, it is important to highlight that hydrosilylation has been extensively utilized to selectively produce silyl enol ethers or silyl enolamines without the need for a base. A particularly noteworthy study investigated the selectivity of this reaction when imines were treated with different silanes in the presence of a set of ruthenium thiolato complexes including **9a** (already reported for the TH of ketones) (Ohki et al., 2008b) and **9c** (Hermeke et al., 2014). The authors reported that treating enolizable *N*-benzyl imine with complexes **9a**,**c** resulted in a markedly different product distribution compared to reactions involving *N*-phenyl imines. Reaction rates were found to depend significantly on the steric and electronic characteristics of the Ru–S bond: electron-rich phosphines enhanced reactivity, while increased steric bulk generally slowed the reaction (Scheme 16). High chemoselectivity toward silyl enolamine formation was typically observed when the reaction was conducted under open conditions, allowing for the release of dihydrogen. However, both catalysts **9a** and **9c** yielded *N*-silylated amines when *N*-benzyl imines were used as substrates. This shift in selectivity was attributed to the lower steric hindrance of the imine and the reduced acidity at the *α*-position. Additionally, the nature of the phosphine ligand played a critical role: the bulky, electron-withdrawing phosphine in complex **9c** led exclusively to the formation of *N*-silylated amines (Scheme 16).

**SCHEME 17:**
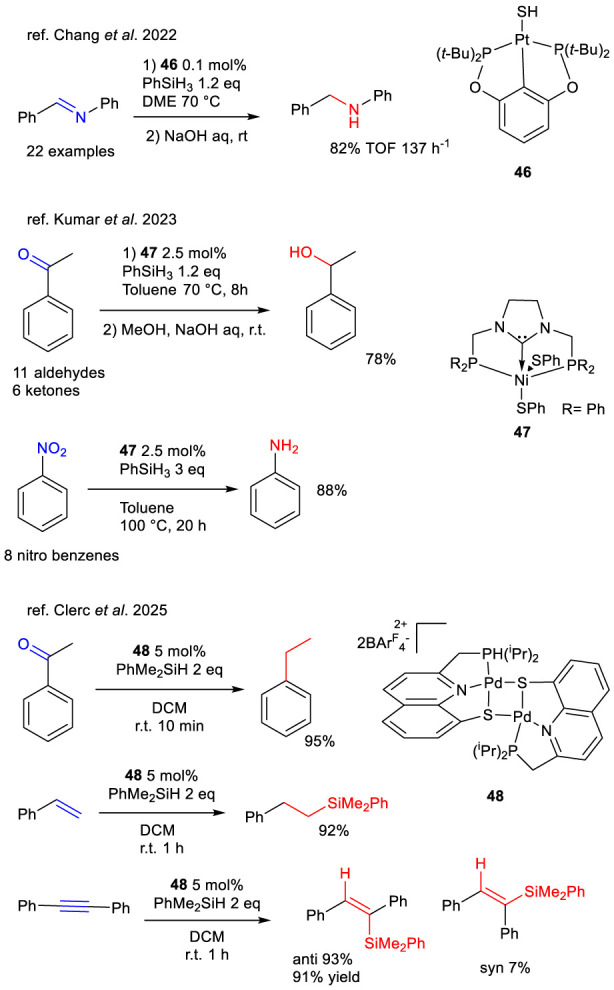
Silane-promoted TH of imines, ketones and nitroarenes catalyzed by complexes 46–48 and hydrosilylation of C–C multiple bonds catalyzed by complex 48.

In addition to ruthenium, palladium, platinum, and nickel pincer thiolato complexes have also been investigated for TH using silanes (Scheme 17). Among them, Pt (II) thiolato complexes (e.g., complex **46**) emerged as the most efficient transition metal catalysts for the hydrosilylation of aldimines, achieving a TOF of 137 h^-1^ in reactions with *N*-benzylidenaniline (Chang et al., 2022). The study demonstrated not only the high catalytic efficiency but also the broad substrate scope, with successful application to 22 imine substrates.

A pincer carbene nickel complex was also effectively employed by Kumar et al. (2023) in the TH of benzaldehydes, ketones, and nitrobenzene using phenylsilane. The nickel thiolato complex **47** outperformed its chloride and hydride counterparts, underscoring the pivotal role of the thiolate ligand in promoting Si–H bond activation via MLC. This mechanism parallels that of the Pt (II) complex **46**, which similarly showed superior performance compared to its hydride analogue in the presence of a thiolate ligand (Scheme 17).

A recent strategy further explored Pd/S cooperative catalysis for hydrosilylation, with the dimeric catalyst **48** demonstrating high efficiency in the TH of alkynes (Clerc et al., 2025). The reaction exhibited excellent regio- and stereoselectivity, favoring *β*-addition of the silane group. This catalyst also offered enhanced solubility, stability, and exclusive selectivity for hydrosilylation over dehydrogenative coupling, thus overcoming limitations commonly associated with ruthenium-based systems (Scheme 17).

An alternative of interest is described by Baker and colleagues, who report SNS-type pincer catalysts containing the non-noble metals Cu and Zn.

**SCHEME 18:**
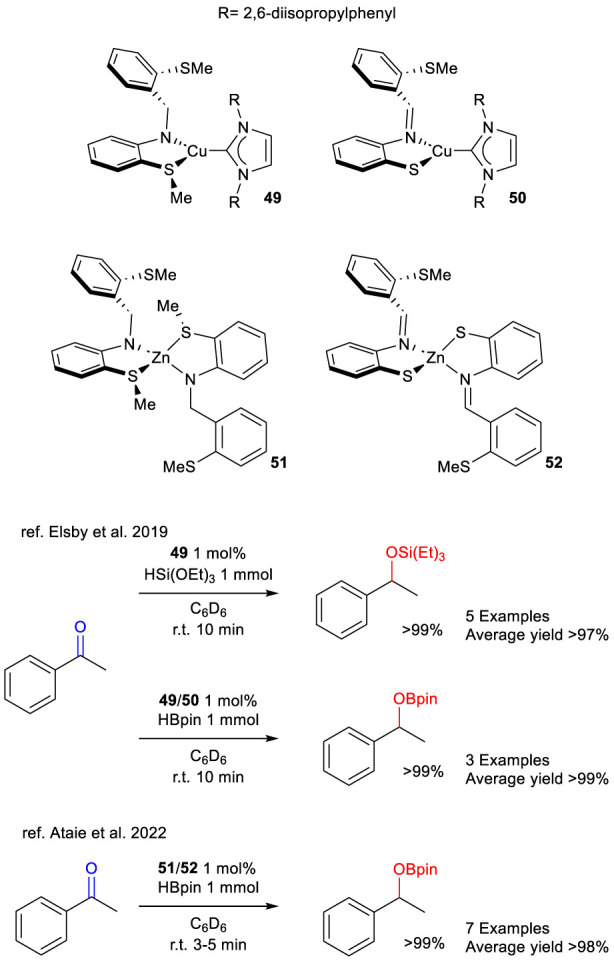
Hydrosilylation of acetophenone mediated by complex 49 and hydroboration of acetophenone mediated by complexes 49–52.

In 2019, Elsby and Baker (2019) introduced catalysts **49** and **50**, which were based on Cu(I) and an NHC carbene ligand (Scheme 18). These complexes differ by a thioether group acting as a neutral ligand, and a thiolate group functioning as an anionic ligand, respectively. As previously described, sulfur atom is directly involved in the activation and subsequently splitting of Si-H bond in the case of hydrosylilation. The same behavior is to be expected in the case of B–H bond which occurs in the case of hydroboration, thus such sulfur bearing complexes could be involved in both reactions. Indeed, complex **49** is reported to be active under hydrosilylation conditions using triethylsilane under mild reaction conditions, whereas both complexes **49** and **50** are reported to be active in hydroboration conditions employing pinacolborane under mild conditions. This study offers a rare illustration of the successful application of a non-noble metal, such as copper, in the context of TH in the presence of a sulfur-based ligand.

In 2020, Ataie et al. (2022) reported on Zn(II)-based SNS pincer catalysts bearing either a thioether group (complex **51**) or a thiolate group (complex **52**). It has been reported that both complexes are active under hydroboration conditions employing pinacolborane, operating under mild conditions, and exhibiting shorter reaction times compared to the copper analogues **49** and **50** (Scheme 18). This study offers a noteworthy illustration of the effective utilization of zinc, a non-noble metal, in conjunction with a sulfur-based ligand in such transformations.

## 4 Enantioselective (transfer) hydrogenative processes

The mounting demand from industries such as the pharmaceutical sector, where the production of enantiomerically pure molecules is paramount, has propelled research towards the development of highly enantioselective processes. This objective has been fully realized with the advent of highly active and selective chiral organometallic catalysts, a breakthrough that earned Profs. Sharpless (enantioselective oxidation), Knowles and Noyori (enantioselective reduction) the 2001 Nobel Prize in Chemistry. These catalysts, with their inherent chiral information embedded in their three-dimensional structure, have been demonstrated to effectively control the enantioselectivity of chemical transformations. The incorporation of optically pure chiral ligands during the synthesis of the complexes serves to introduce the requisite chiral information. The three-dimensional arrangement of these ligands compels the substrates to approach the catalytic site in a highly specific and consistent manner, analogous to the mechanism by which enzymes operate in nature.

Sulfur has been the focus of considerable research due to its electronic properties, which permit its incorporation into a broad array of functional groups. This element has been the subject of investigation for its potential application in the development of chiral ligands suitable for use in the enantioselective reduction of prochiral substrates. It should be noted that there are numerous examples of thioamide-(Coll et al., 2014; Zaitsev and Adolfsson, 2006), sulfonamide-(Liu et al., 2022; Zhang et al., 2023; Zhao et al., 2022) and sulfinamide-(Zani et al., 2008) based ligands in which no strong evidence that the sulfur atom is bound to the metal core emerges; therefore, these cases will not be further discussed.

The hemilability of the sulfur-metal bond has posed significant challenges in the process of crystallizing these chiral complexes. Indeed, the majority of instances in which this system has been successfully employed in (transfer)hydrogenative processes are characterized by the *in-situ* formation of the active species from the precursors.

The earliest efforts to incorporate sulfur into chiral ligand frameworks focused on bidentate SN-type ligands. These systems have been shown to generate the active catalytic species *in situ* through reaction with the metal precursor, and they have been examined extensively in the TH of ketone substrates. As discussed below, stereocontrolled HY using sulfur-based catalysts can be achieved with both pincer complexes and half-sandwich complexes. Although pincer complexes possess well-defined and relatively rigid structures, sulfur-containing ligands have been studied more extensively in half-sandwich architectures. Therefore, for clarity, the catalysts are categorized according to catalyst type, as no approach has yet demonstrated a clearly superior influence on the reaction outcome.

**SCHEME 19:**
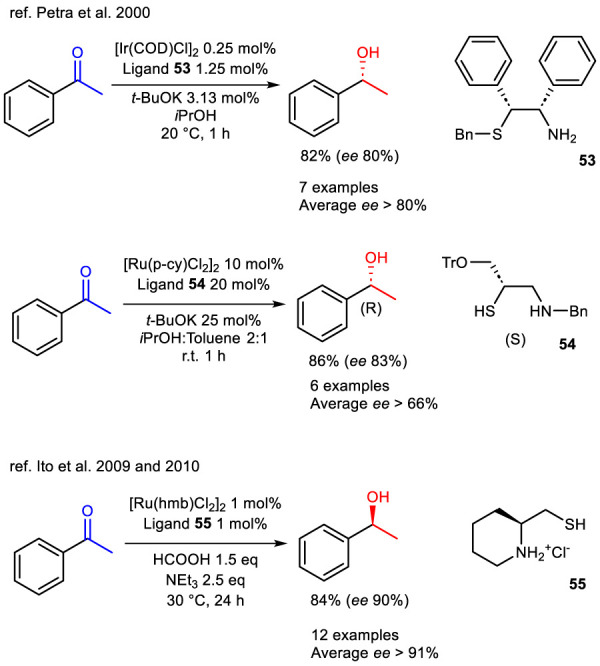
Enantioselective TH of ketones to optically active carbinols catalyzed by *in situ* generated systems from Ir- and Ru-precursors and ligands 53, 54 and 55, respectively.

Chiral iridium and ruthenium SN-type ligand catalysts were introduced in the early 2000s for their potential in the enantioselective reduction of acetophenone. Petra et al. (2000) reported an iridium–aminosulfide complex, while Harfouche et al. (2004) proposed a ruthenium–aminoethanethiol catalyst. In both studies, several ligands synthesized by van Leeuwen’s group were evaluated, with those containing a thioether moiety demonstrating superior performance compared to their sulfoxide-bearing counterparts (Petra et al., 2000). Among these, ligand **53** emerged as the most active derivative, delivering an enantiomeric excess (ee) of 80% in favor of the *R*-enantiomer in the TH of acetophenone (Scheme 19).

The ruthenium catalyst was found to be less efficient, requiring 10 mol% loading in *i*PrOH/toluene. However, in the presence of ligand **54**, 83% ee toward the *R*-enantiomer was achieved (Scheme 19). The manuscript also detailed that ligand **54** was synthesized efficiently from the chiral starting material (*R*)-trityl (thiiranylmethyl) ether via a concise two-step synthetic route.

Another example of thiol-based chiral SN ligands is reported in the works of Ito et al. (2009) and Ito et al. (2010), which describe the synthesis and application of 2-aminoethanethiol ligands in the formation of dinuclear ruthenium *η* ^6^-hexamethylbenzene ([Ru (hmb)Cl_2_]_2_) complexes. The most efficient complex reported is that formed with ligand **55**, which enabled the selective formation of the *S*-enantiomer with a 90% enantiomeric (Scheme 19). However, it should be noted that this protocol is accompanied by two significant limitations. Firstly, it necessitates the use of triethylamine as the solvent and the base. Secondly, it requires relatively long reaction times.

While the approach based on aminothiol ligands proved less effective when applied to asymmetric (transfer) hydrogenation protocols and was therefore soon abandoned, the development of aminothioether-based ligands suitable for these protocols has continued and culminated in the emergence of SNNS-type catalytic systems in the following years.

**SCHEME 20:**
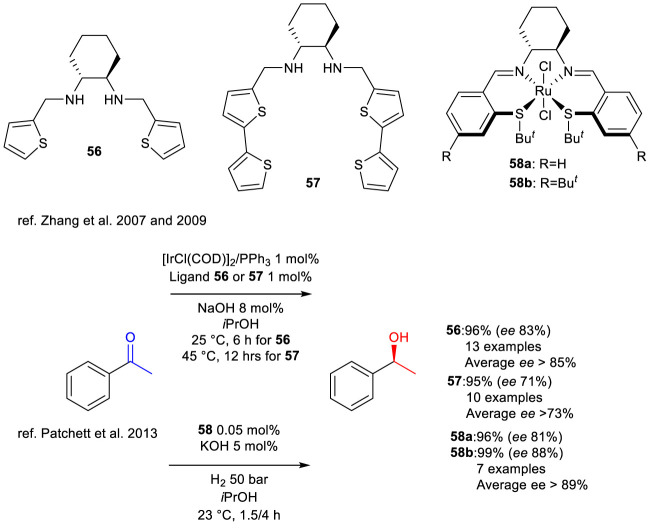
Enantioselective TH of ketones to optically active carbinols promoted by *i*PrOH and catalyzed by *in situ* generated system [IrCl(COD)]_2_/PPh_3_/56,57 and by complexes 58a,b.

In the studies conducted by Zhang X. Q. et al. (2007) and Zhang et al. (2009), the synthesis of chiral diamino-thiophene ligands **56** and **57** was described, along with their use *in situ* to generate an iridium-based catalytic system effective in the TH of aromatic ketones. The research group highlighted the importance of the nitrogen atom in its amine form for the efficiency of the systems for the targeted transformation, whereas its presence in the imine form has a detrimental effect on such systems. The active form of ligand **56** enabled the efficient conversion of acetophenone to the *S*-enantiomer of 2-phenylethanol with an enantiomeric excess of 83% under mild reaction conditions (Scheme 20) while the active form of ligand **57**, probably for the excessive steric hindrance of the two non-chelating thiophene groups, required a higher temperature and a longer reaction time in order to perform the desired transformation.

In the 2013 study by Patchett et al. (2013), the synthesis of several chiral SNNS ligands featuring a thioether-based structure is reported, along with their application in the synthesis of ruthenium complexes that are active in the enantioselective HY of both aromatic and aliphatic ketones. The authors identified complexes **58a** and **58b**, in which nitrogen is present in its imine form, as the most active. The three-dimensional structure of complex **58a** was confirmed through X-ray crystallography. Utilizing these complexes, the *S*-enantiomer of 2-phenylethanol was successfully synthesized from acetophenone, yielding an enantiomeric excess exceeding 81% under highly mild reaction conditions (Scheme 20). Furthermore, the authors stated that the amount of hydrogen used could be reduced from the optimal conditions of 30 bar.

Another intriguing approach, which builds upon the 1997 work by Barbaro et al. (1997) and in the 2007 work of Le Roux et al. (2007) (in which various chiral thioether-based PS-type ligands were synthesized and employed for the formation of organometallic complexes), involves the development of mixed chiral PNS ligands. These ligands maintain a phosphine group within their structure, in analogy with previously described complexes active in catalytic HY processes.

**SCHEME 21:**
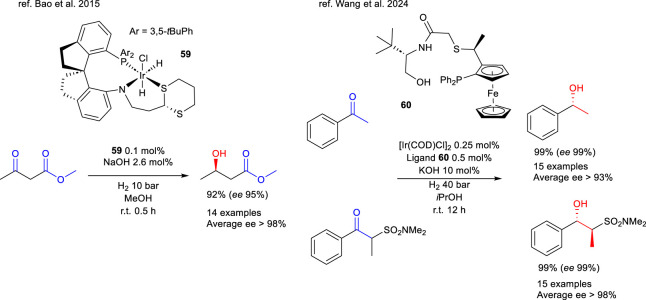
Enantioselective HY of *β*-ketoesters, ketones and *α*-alkyl-*β*-ketosulfonamides catalyzed by complex 59 and by the *in situ* generated system [IrCl(COD)]_2_/60.

In 2015, Bao et al. reported the synthesis of several PNS-type ligands, characterized by the presence of a spiro group in their carbon backbone (Yang et al., 2023), and described their use in the synthesis of iridium complexes (Bao et al., 2015). However, these complexes could not be crystallized, and their structures were therefore investigated through NMR spectroscopy. Notably, complex **59**, featuring sulfur in the form of a thioacetal, exhibited remarkable activity in the enantioselective HY of the carbonyl group in *β*-alkyl-*β*-ketoesters or amides. The research group did not report any differences between protocols employing either the pre-isolated catalyst or the *in situ* generated one while it is notable that the PNS complexes resulted more active than the PNP analogues previously described by the research group. In the case of methyl acetoacetate HY, the *R*-enantiomer was obtained with a 95% enantiomeric excess using a very low catalyst loading and remarkably short reaction times (Scheme 21). Subsequent studies by the research group expanded the reaction scope of complex **59** and investigated its application in dynamic kinetic resolution (Wu et al., 2024; Yang et al., 2021).

A recent example of tridentate PNS ligands applied to asymmetric processes is reported in the 2024 work by Wang et al. (2024) This study describes the synthesis of chiral thioether ligands containing an amide substructure, their use for the *in situ* generation of iridium catalytic species, and their investigation in the HY of ketone substrates. Ligand **60** was identified as the most active and enantioselective derivative, and its application to structurally diverse ketones and *α*-alkyl-*β*-ketosulfonamides was examined, revealing high selectivity towards the ketone functional group. In the case of acetophenone, the use of ligand **60** enabled the synthesis of the *R* enantiomer with excellent yield and enantiomeric excess (Scheme 21).

**SCHEME 22:**
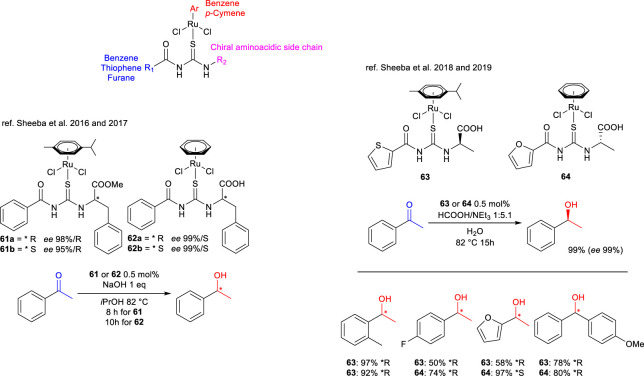
Enantioselective TH of ketones to optically active carbinols promoted by *i*PrOH and catalyzed by 61,62 or promoted by HCOOH/NEt_3_ and catalyzed by 63,64.

One of the most selective catalytic systems, and certainly the one that has undergone the most intensive development, involves the study of ruthenium complexes bearing a monodentate thiourea ligand. These complexes are applicable under TH conditions. These systems have been described in the works of Sheeba et al. since 2014, detailing the synthesis of chiral ligands derived from commercially available enantiopure amino acids. These ligands exhibit notable differences in several aspects: the nature of the *η* ^6^-coordinating group (benzene or *p*-cymene), the nature of the aryloyl substituent R_1_ (benzene, furan, or thiophene), and the structure of the side chain R_2_, which depends on the chiral amino acid used, as illustrated in Scheme 22.

The structure of most of the complexes described in these studies has been confirmed by X-ray crystallography, revealing that they are relatively stable and readily crystallizable. This evidence supports the hypothesis that the thioureidic sulfur functions as a monodentate ligand, superseding the role of the nitrogen atoms (Scheme 22).

Due to the substantial number of complexes developed and the observation that their enantioselectivity is not strictly contingent on their structural variations, only the complexes that have yielded optimal results will be discussed.

The studies published on 2014 (Sheeba et al., 2014) and on 2015 (Mary Sheeba et al., 2015) represent preliminary investigations of this catalytic system, which led to the development of complexes **61a-b**, reported in the 2016 study (Sheeba et al., 2016), and complexes **62a-b**, described in the 2017 study (Sheeba et al., 2017). A distinguishing feature of both categories of complexes is the use of benzene as the R_1_ substituent, with *R*- or *S*-phenylalanine serving as the amino acid-derived side chain. The primary distinction between the two categories lies in their *η* ^6^-coordinating group, with *p*-cymene being utilized in complexes **61a-b** and benzene being employed in complexes **62a-b**. These complexes have been identified as the most active and enantioselective derivatives reported by the research group, exhibiting excellent conversion rates and enantioselectivity (Scheme 22). However, the observed enantioselectivity appears to depend more on the overall three-dimensional structure of the complexes rather than on the configuration of the chiral center in the ligand. For instance, acetophenone is preferentially converted into the *R* enantiomer when complexes **61a-b** (with either *R* and *S* ligand configurations, respectively) are utilized, and the *S* enantiomer is preferentially obtained when complexes **62a-b** (Scheme 20) are employed. Furthermore, it is to be noted that complexes **62a-b** bearing a benzene as *η* ^6^-coordinating group performs faster compared to their *p*-cymene counterparts.

These complexes have been selected as the optimal model; however, it is imperative to acknowledge that all other complexes described in the aforementioned studies exhibit catalytic activity within the defined protocol, with enantioselectivity ranging from moderate to excellent. This selectivity is not solely determined by the ligand’s structure but rather by the combined influence of the ligand and the *η* ^6^-coordinating group.

Given the ease with which the structure of these complexes can be modified simply by changing the amino acid used during synthesis and considering the generally good water solubility of amino acids and small peptides, the research group directed its efforts toward the preparation of water-soluble ruthenium complexes. The objective was to develop catalytic systems that were active in TH in aqueous media, representing a greener evolution of the process. This objective was accomplished by employing D- or L-alanine as the amino acid, a strategy that involved the removal of the bulky and lipophilic benzene group. In 2018 (Sheeba et al., 2018), the authors reported complexes featuring benzene as the *η* ^6^-coordinating group, while in 2019 (Sheeba et al., 2019), they described analogous complexes bearing *p*-cymene. To the best of our knowledge, this represents a unique case of organosulfur ligand-based catalysts being successfully employed in asymmetric TH in water.

In this instance as well, the derivatives with *η* ^6^-benzene were found to be the most rapid, while complexes **63** and **64**, distinguished by the heterocyclic aryloyl moiety, exhibited the greatest activity and enantioselectivity in the TH protocol of acetophenone in water, employing formic acid as the hydrogen donor and triethylamine as the base. Their versatility was further confirmed by testing various aryl ketones as substrates (Scheme 22).

As demonstrated by the findings, the efficiency and enantioselectivity of complexes active in asymmetric processes are contingent not only on the three-dimensional structure of the catalyst, but also to a significant extent on the structure of the substrate. The outcomes demonstrate variability depending on the steric hindrance and electronic characteristics of the carbonyl group. Consequently, while the complexes reported in the literature are identified as the most active and selective for acetophenone, it is crucial to acknowledge the significance of the other complexes described by the authors, as their use may be complementary depending on the substrate under investigation.

**SCHEME 23:**
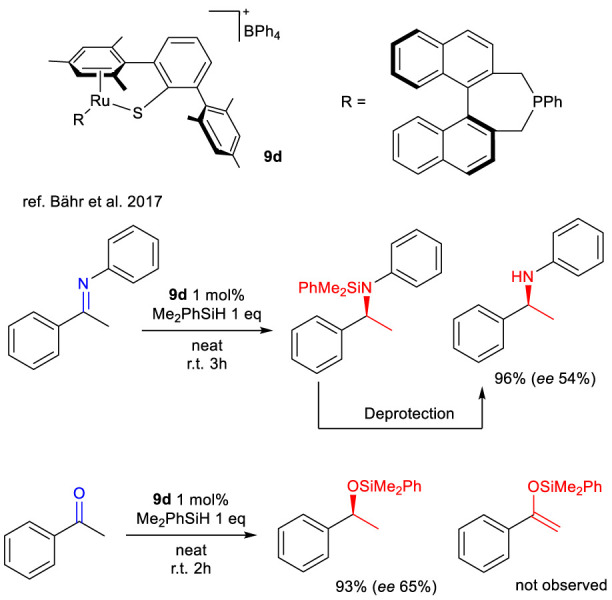
Dehydrogenative silylation of imines and ketones with silanes catalyzed by complex 9d.

Bähr and Oestreich (2017) made a significant contribution to the field of enantioselective hydrosilylation by reporting on complex **9d**. This is an analog of complexes **9a**–**c**, which were previously described. It features an asymmetric phosphine ligand that gives the system its chirality. The researchers tested complex **9d** in the enantioselective hydrosilylation of (*E*)-*N*,1-diphenylethan-1-amine and acetophenone under the conditions summarized in Scheme 23.

For imines, the reaction produced (*S*)-*N*-(1-phenylethyl)aniline with a high yield and *ee* of 54% after 3 hours. For acetophenone, the corresponding (S)-silyl ether was obtained in high yield after 2 hours with *ee* of 65%. Various silyl hydrides were tested as hydrogen sources. With the exception of Ph_3_SiH and PhSiH_3_, enantioselectivity showed little dependence on the type of hydride reagent used. Me_2_PhSiH was the most effective due to its result to higher yields.

Previous studies on complexes **9a** and **9c** revealed that the primary products of the hydrosilylation of imines were *N*-silyl enamines. The authors attributed the markedly different outcome of **9d** to the absence of H_2_ removal from the reaction environment. This allowed the reaction to naturally proceed toward forming the corresponding amine.

Though the obtained enantiomeric excess is moderate, this work is a rare example of asymmetric catalysis in which a single chiral monodentate phosphine ligand coordinating to the metal center is responsible for enantioinduction. Despite the modest *ee* values, this remains the first example of enantioselective net C = X hydrosilylation involving cooperative Si–H bond activation.

## 5 Borrowing hydrogen

**SCHEME 24:**
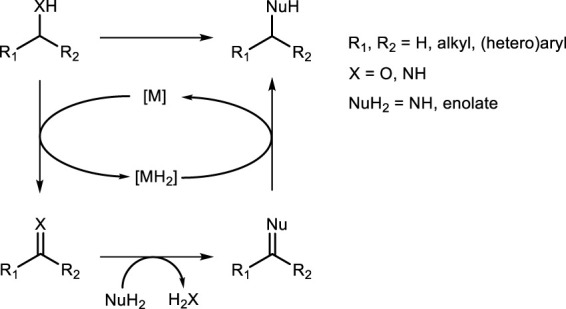
Representation of a generic borrowing hydrogen process mediated by a transition metal complex.

Catalytic borrowing hydrogen (BH) approach (also known as hydrogen auto-transfer) is a relatively recent strategy for implementing highly important organic transformations (Scheme 24). A great advantage of the catalytic BH is the elimination of hazardous or toxic stoichiometric reagents, thus resulting in a benign and environmentally responsible approach. For these reasons, a large number of catalytic systems (especially homogeneous) have been designed and extensively been studied in the past 2 decades (Reed-Berendt et al., 2021). In this context, transition metal complexes bearing sulfur-based ligands as active catalysts in BH processes have emerged and attracted great attention within the scientific community (Guillena et al., 2007). BH approach is also of great importance because it involves the formation of new C–N (if NuH_2_ is an amine) and C–C (if NuH_2_ is an enolate anion) bonds (Scheme 24).

**SCHEME 25:**
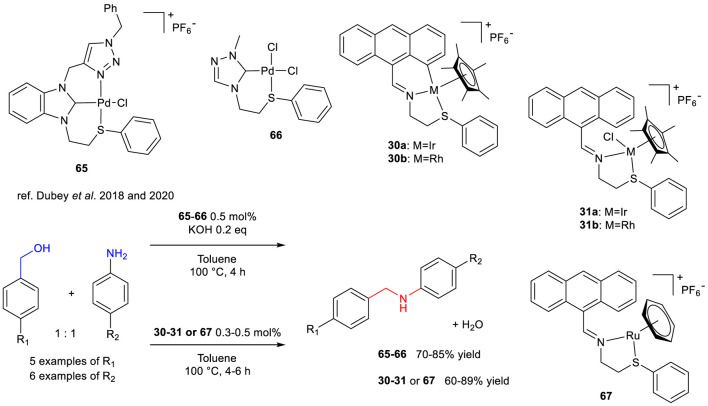
*N*-Benzylation of aniline with benzyl alcohol catalyzed by 65, 66 or 30, 31 and 67.

Dubey and Singh reported that two Pd(II)-complexes **65** and **66** bearing bidentate NHC-thioether ligands were active in the *N*-benzylation of aniline and 4-substituted derivatives, employing benzyl alcohol and 4-chloro, 4-methyl and 4-methoxybenzyl alcohols (Scheme 25). The optimized reaction conditions were 100 °C in toluene, KOH (20 mol%) and the chosen Pd complexes (0.5 mol%) over 4 h. The achieved conversions and isolated yields of the BH products ranged from good to excellent (Scheme 25, 79%–96% and 70%–85%, respectively). The time profile of the *N*-benzylation of aniline catalyzed by complex **65** indicates that 60% conv. Was reached after 2 h of reaction, achieving almost complete conversion after 4 h. Recyclability tests (up to 9 runs), showed unchanged catalytic activity after 6 cycles and gradual drop till the ninth run (down to 45% conv.). Although the limited scope of the *N*-benzylation process, the authors have proven that Pd(II)-complexes featuring mixed NHC-thioether may be used as promising pre-catalysts for BH reactions (Dubey and Singh, 2020).

The same authors also reported that Ir(III)-, Ru(II)- and Rh(III)-complexes (Dubey et al., 2018; 2019) **30**,**31** (already reported for TH of carbonyl compounds) and **67**, bearing an easily achievable bidentate phenyl-thio imine ligand were active in both TH and BH reactions. The latter was explored with diverse benzyl alcohols as *N*-alkylating agents for amino arenes, using toluene (100 °C) low catalyst loadings in all cases (0.3–0.5 mol%) and relatively short reaction times (4–6 h) as the optimized reaction conditions. The isolated yields were gratifying (60%–89%), showing general lower productivity with electron-deficient 4-fuoroaniline and slightly higher average activity and robustness with Rh- and Ir-complexes bearing bidentate ligands **31a**-**b** with respect to their cyclometallated congeners **30a**-**b** (Scheme 25). Ru-complex **67** showed an average lower activity and productivity with respect to its Rh- and Ir-congeners (Scheme 25). Although the scope is limited to a low number of substrates, it is worth mentioning that this class of complexes were able to mediate the BH process without the additional use of an alkali base, which is often required for the generation of catalytically active metal hydride species. The reusability of the best Ir(III)-complex was tested in the *N*-benzylation of aniline up to six runs, showing a progressive detriment of the catalytic activity after every reuse, thus indicating a gradual decomposition/deactivation of the complex during time.

**SCHEME 26:**
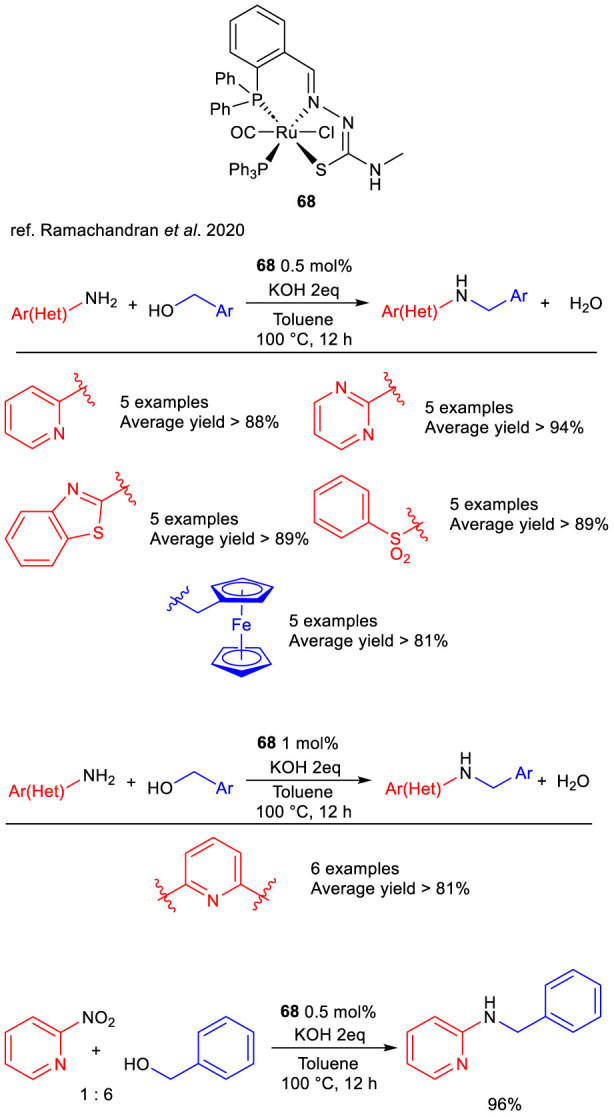
*N*-Alkylation reactions catalyzed by complex 68.

Ramachandran et al. and co-workers studied the activity of tridentate Ru(II)-PNS complexes in the *N*-alkylation of aminoheterocycles, with PNS being thiosemicarbazone-based ligands in which the sulfur atom coordinates the Ru center as anionic sulfide during complexation. After a detailed study on 2-aminopyrdine and benzyl alcohol, complex **68** bearing an *N*-methylated thiosemicarbazone ligand was found the most active under the optimized reaction conditions (0.5 mol% [Ru], 2 eq. of KOH, 100 °C in toluene, 12–24 h), reaching >99% yield. Pre-catalyst **68** demonstrated to efficiently mediate the *N*-benzylation of aminopyrimidine, aminobenzothiazol and sulfonamide with diverse 4-subsituted benzyl alcohols (Scheme 26, 76%–97% yields). Also, 2-amino pyridine and 4-substituted aminoarenes were successfully reacted with ferrocenyl carbinol to give the corresponding alkylated products from good to excellent yields (Scheme 26, 68%–91%). 2,6-Diaminopyridine underwent double *N*-alkylation with different primary alcohols (Scheme 26, 75%–93%). It is worth noting that *N*-benzyl-2-amino pyridine could be obtained in excellent yield (Scheme 26, 96%) from the more readily available 2-nitro pyridine in the presence of a six-fold excess of benzyl alcohol as hydrogen source and complex **68**, entailing a tandem TH/BH sequence. Interestingly, the nitro group on the pyridine ring underwent reduction to amine via TH, indicating this type of complexes as promising pre-catalysts for different hydrogenative process (Ramachandran et al., 2014).

**SCHEME 27:**
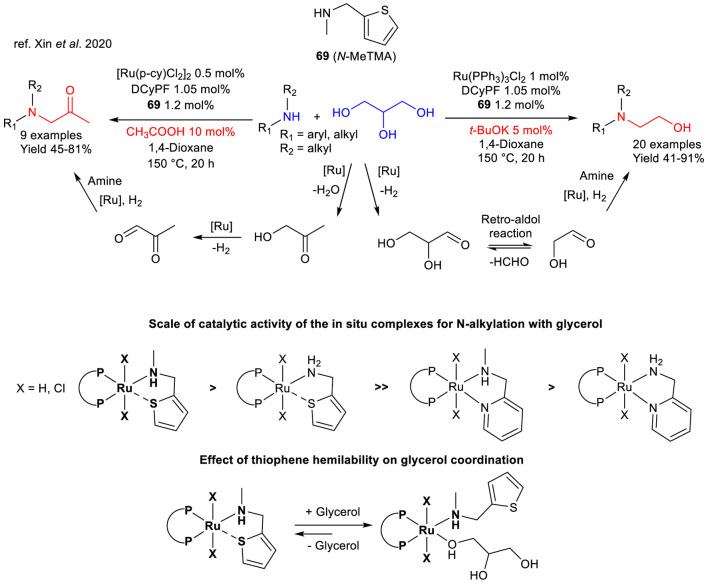
Acid/base modulation of *N*-acetonylation and *N*-hydroxyethylation of secondary amines using glycerol as unique alkylating agent catalyzed by Ru(II)-complexes/*N*-MeTMA, catalytic activities of the main complexes employed in the study and effect of the thiophene moiety.

An interesting example recently reported by Li and co-workers showed that *in situ* generated complexes from commercially available Ru(II)-precursors, 1,1′-bis(di-cyclohexylphosphino)-ferrocene (DCyPF) and thiophene-based ligands were able to catalyze two different *N*-alkylation reactions of secondary amines using glycerol as alkylating agent in both cases. The two reactions were found to be dependent on the acid-base properties of the additive employed. Among the several amino-based ligands used, *N*-methyl-2-thienylmethyl amine (*N*-MeTMA **69**) resulted as the best fit to achieve efficient BH process. As a matter of fact, when a catalytic amount of a strong alkali base was used (5 mol%), *N*-hydroxyethylation was observed, whilst in the presence of acetic acid (10 mol%), the preferred reaction pathway led to the obtainment of the *N*-acetonylation product (Scheme 27). On the other hand, primary amines such as aniline and benzylamine were not found to be suitable substrates for this process, leading to complex mixtures, probably due to their higher reactivity with respect to secondary amines (Xin et al., 2020).

Mechanistic investigations performed by NMR spectroscopy under both basic and acidic conditions suggested that in the former case retro-aldol reaction occurred at glyceraldehyde to give the key intermediate for BH to achieve hydroxyethylation. On the other hand, under acidic conditions, glycerol underwent dehydration to give hydroxyacetone which led to *N*-acetonylation via BH (Scheme 27). In addition, the Authors suggested that the higher activity of the SN ligand compared to its congeners such as 2-aminomethyl pyridine, it can be inferred that the hemilability of the thiophene moiety may favor the optimal glycerol coordination and activation for the BH process, apparently favoring its monocoordination with respect to a bidentate fashion (“poisonous chelation effect”) (Scheme 27). This work proved that under these two “switchable” BH strategies, highly functionalized tertiary amines may be obtained from secondary amines and highly available biomass-derived glycerol.

An intriguing instance of sulfur-based organometallic complexes, alternatively to the previously documented ones, is presented in the research of Maji and colleagues. These researchers describe phosphine-free Mn SNN complexes that exhibit activity a wide variety of BH reactions. These are manganese complexes **70** and **71**, distinguished by the presence of a thiophene and a thioether sulfur atom respectively.

**SCHEME 28:**
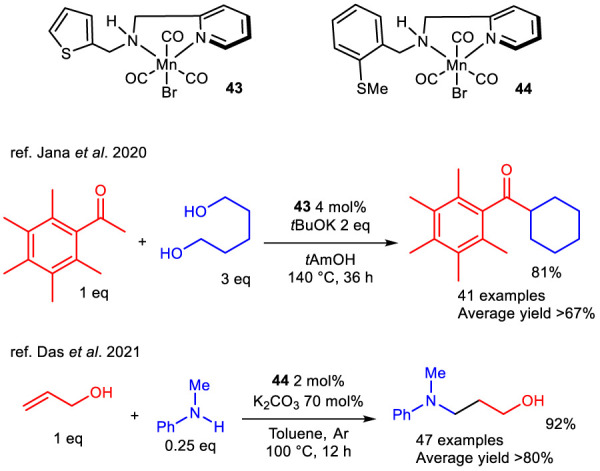
BH reactions catalyzed by Mn-based catalysts 43 and 44.

In their 2020 work, Jana et al. (2020) reported the activity of complex **43** under double α-alkylation conditions for the stereoselective synthesis of (1 + n)-membered cycloalkanes. This was achieved through the reaction between aryl ketones and terminal diols in the presence of *t*-BuOK. The reaction proceeds through two sequential cycles of alcohol DHY/aldol condensation/C=C HY, in order to doubly functionalize through a hydrogen auto-transfer process the only free α-position of aryl ketones (Scheme 28).

Also, in 2021, Das et al. (2021) reported the activity of complex **44** in the anti-Markovnikov hydroamination of allyl alcohols. In this process, a primary amine is treated in the presence of an excess of allyl alcohol to form a new σ-type C-N bond. The reaction proceeds through alcohol DHY, followed by a Michael addition and a final aldehyde HY with the manganese catalyst mediating the auto-transfer process of hydrogen on the same substrate (Scheme 28).

The catalytic cycle for **43** and **44** is supposed to proceed via sequential decarbonylation/sulfur coordination on the Mn-center, although this phenomenon is postulated from computational outcomes.

## 6 Mechanistic insight into the cooperative effect of thiolate and thioester in hydrogenation and transfer hydrogenation

The synergistic participation of a metal and its ligand (metal ligand cooperativity, MLC; see Figure 1) has emerged as a powerful strategy in modern catalysis. Significant effort has been devoted to rationalizing the cooperative role of sulfur-based ligands in (de)hydrogenative transformations, in which the ligand directly participates in bond activation, undergoes reversible chemical changes, and becomes an integral component of the reactive site. While the contribution of catalysts featuring polar metal–nitrogen bonds to HY chemistry is well documented, typically involving bond activation across a Lewis-acidic metal center and an adjacent Lewis-basic ligand (Zhao et al., 2013), detailed kinetic and mechanistic studies for systems employing metal–sulfur cooperation remain comparatively scarce.

The potential of coordinated thiolate donors to function as Brønsted bases was first recognized in 1993 by Mathis Moll and co-workers (Sellmann et al., 1993) inspired by the crucial roles played by polar metal–sulfur motifs in enzymatic processes such as H_2_ heterolysis in hydrogenases. Subsequent advances further cemented the viability of sulfur-assisted MLC: in 2008, Ohki and Tatsumi reported the spectroscopic and crystallographic characterization of the dihydrogen addition product of an iridium thiolato complex, providing clear evidence for thiolate cooperativity (Ohki et al., 2008a) (Scheme 28). In 2009, Seino and Mizobe demonstrated the heterolytic cleavage of H_2_ by rhodium thiolato complexes (Misumi et al., 2009).

**SCHEME 29:**
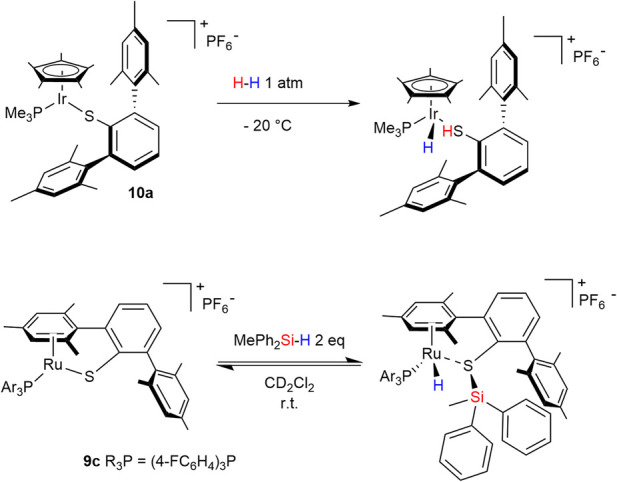
Mechanism of thiolate-based MLC for H-H and Si-H activation.

More recently, the application of sulfur-based MLC has been successfully extended to the heterolytic activation of Si–H and B–H bonds, granting access to silicon and boron electrophiles with compelling experimental support. The heterolysis of Si–H bonds is inherently more feasible than that of H–H bonds due to their greater bond length and lower bond dissociation energy; in this context, the cooperative action of the ligand becomes essential for stabilizing and activating the silicon electrophile. Unlike harder N- or O-donor ligands, soft sulfur donors offer distinct advantages, a theme explored extensively in Section 3.2. The mechanistic underpinnings of this reactivity were conclusively established by Stahl and co-workers in 2015 (Stahl et al., 2015) (Scheme 29).

The influence of thioether donor strength, coordination geometry, and the overall ligand environment on the activity and selectivity of (de)hydrogenative processes still requires much more systematic investigation. Owing to the potentially hemilabile character of sulfur donors, these ligands combine hemilability with the behavior of Lewis-basic “actor” ligands, which complicates mechanistic studies (Bassetti, 2006).

Therefore, despite the considerable interest in the application of thioether- and thiophene-based ligands in HY processes, the debate regarding their mechanistic implications remains open and, in some cases, contradictory (Chen et al., 2016; Matiello et al., 2025; Spasyuk et al., 2013).

**FIGURE 4:**
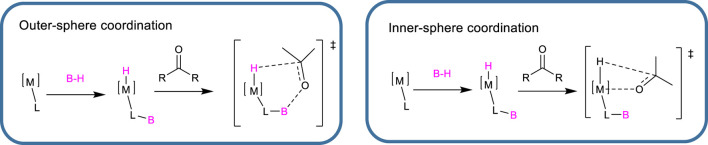
Principal mechanistic pathways for sulfur-based metal complexes.

Considering of TH of carbonyl compounds, several approaches have been employed to examine this mechanism in the presence of thioether- or thiophene-ligated metal complexes and B–H or Si–H reagents and two principal mechanistic pathways are generally proposed: a) an outer-sphere transition state, and b) an inner-sphere transition mechanism (Figure 4).

In pathway (a), MLC plays a major role, arising from the direct involvement of the basic ligand. In pathway (b), the hemilabile character of the sulfur ligand may positively influence the process by enabling dynamic coordination modes.

Because a comprehensive discussion of all mechanistic possibilities is beyond the scope of this work, two representative examples are presented to illustrate the significance of thioether in preactivation of the catalyst.

**FIGURE 5:**
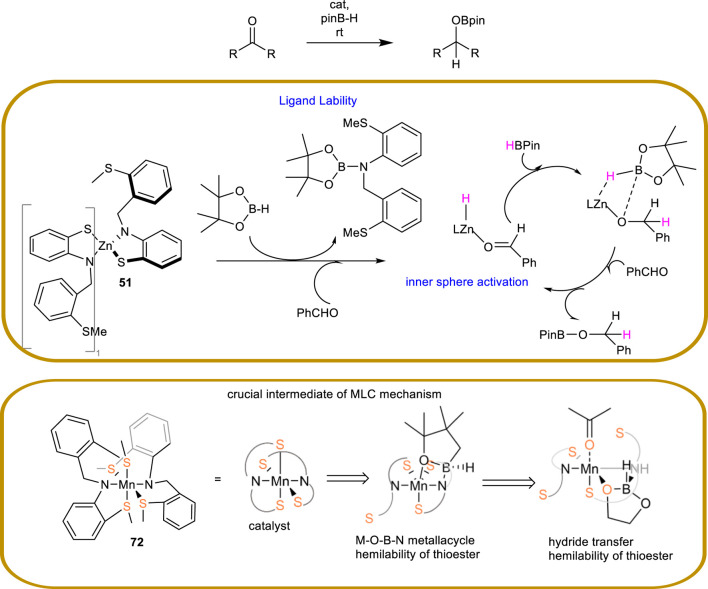
Mechanism of thioester-based MLC for hydroborination reaction with catalysts.

In 2021 and 2022, detailed mechanistic studies on carbonyl hydroboration catalyzed by Zn **51** and Mn **72** complexes were performed by means of density functional theory (DFT) calculations and reported by the Tom Baker group (Ataie et al., 2022; Elsby et al., 2021). These studies revealed that both systems undergo ligand-assisted precatalyst activation to generate the catalytically active species. As illustrated in the Figure 5, the proposed mechanism for the Zn catalyst shows that reaction of complex **51** with HBpin results in *N*-borylation of the ligand and formation of a Zn–H species. This Zn–H intermediate rapidly inserts the carbonyl substrate. The mechanism highlights the bifunctional role of the ligand: it activates the B–H bond of HBpin and simultaneously pre-activates the metal center. The Zn–H species is responsible for carbonyl hydride delivery, generating the hydroborated product and being regenerated in the catalytic cycle.

When the same transformation was examined using the Mn catalyst **72**, an unexpected mechanism emerged, supported by DFT calculations (Figure 5). Owing to the hemilabile character of the thioether donor, the reaction does not proceed through formation of a Mn–H species. Instead, a M–O–B–N metallacycle forms, which was calculated to be significantly more stable. The lowest-energy transition state involves carbonyl coordination through the oxygen atom and direct hydride transfer from the N-activated borane to the carbonyl carbon. Although this pathway is inner-sphere, it bypasses a classical metal-hydride intermediate. Computations along the full catalytic cycle highlighted the crucial role of the thioether in stabilizing early intermediates. Initial thioether decoordination and the basic character of the nitrogen donor in activating the borane together demonstrate the multifunctionality of the ligand framework.

## 7 Conclusions and perspectives

This account provides a detailed overview of the most recent and exciting applications of sulfur-based ligands in hydrogenative processes, namely (asymmetric) reduction with molecular hydrogen and TH employing different H_2_-sources, hydrosilylation, hydroboration and borrowing hydrogen processes. Organosulfur compounds of diverse nature (heterocycle, thiourea, thioether and thiolate) have proven to be versatile ligands for stabilizing transition metal centers throughout the catalytic process, most of them belonging to the second and third row. (Asymmetric) HY of carbonyl compounds and imines can efficiently be catalyzed by a large number of Ru, Rh, Ir and Pd complexes bearing thioether and thiolate ligands, although with a limited substrate scope, as well as semi- and total reduction of less reactive esters to hemiacetals and alcohols, respectively. Esters are in some cases (Gusev’s catalysts) hydrogenated to their corresponding alcohols with higher activity compared to their phosphine-based congeners (Firmenich’s, Takasago’s), whereas the former show lower selectivity towards C=C bonds. It is worth pointing out that thioether-based catalysts are also able to perform direct and indirect reduction of CO_2_ to high-added value intermediates (formate salts and amides, respectively), suggesting a great versatility of their catalytic performance. Several thiomamide, thiourea, thioether and thiolate ligands have been explored in the (asymmetric) TH of aldehydes, ketones, and nitroarenes catalyzed by Ru-, Ir- and Pd-complexes, with satisfying enantioselectivities, although their activity and productivity resulted still lower compared with phosphine-based catalysts. Diverse H_2_-sources may be employed, namely 2-propanol and silanes for carbonyl and imine reduction and ethanol/sodium borohydride for the reduction of nitroarenes to their corresponding anilines. Notably, hydridic silanes are also employed in the hydrosilylation of ketones, ketimines and CO_2_, all catalyzed by Ru-thiolato complexes. Fewer examples and fairly limited to thioether ligands are reported on the Ru-mediated borrowing hydrogen process exploiting primary alcohols as alkylating agents for primary and secondary amines and for aryl ketones. In particular, aminothiophene- and aminothioether-based ligands in combination with strongly coordinating phosphines and pyridines, respectively, show that the hemilabile coordination of the thiophene sulfur to the metal center is crucial for the efficiency of the catalysis.

Based on the excellent results of sulfur-based catalysts in (de)hydrogenative processes and on the mechanistic examination, S-donor ligands such as thioethers can be considered a viable alternative to phosphines in both electronic and structural properties. Nevertheless advantages and disadvantages have to be clearly pondered, being the main features reported in the Table 3.

**TABLE 3:** Advantages and Disadvantages of sulfur Ligands.

Advantages of sulfur ligands	Disadvantages of sulfur ligands
*Electronic properties* - Poor *α*-donor and poor *π*-acceptor ligands	*Electronic properties* *-* The metal complex results in higher electron-deficiency relative to its phosphine analogue, often slowing key steps such as oxidative addition, hydride formation, or substrate activation less favorable
*Stability and robustness* - Broad functional-group tolerance - Resistant to oxidation when properly protected/functionalized (e.g., thioether vs. thiolate) - Strong metal–sulfur bonds can enhance catalyst longevity in TH	*Synthetic practicality* - Handling thiols can introduce odor/toxicity concerns
*Reactivity toward metal precursors* - Capable of generating active catalysts *in situ* through ligand-induced activation	*Reactivity toward metal precursors* Less coordinating compared with their phosphine congeners leading to less reactive metal-hydrides
*Ligand flexibility and geometry control* - Enables access to new chiral environments, as coordination of sulfur to the metal can generate a stereogenic center at sulfur - Provides tunable steric and electronic environments that support fine control of enantioselectivity in asymmetric HY (e.g., SN, SNS, and S/N/O pincer architectures)	*Ligand flexibility and geometry control* - Greater conformational flexibility can reduce stereocontrol (low inversion barrier 10–15 kcal/mol) - Difficult to control without multidentate “pincer-like’’ scaffolds
*Activity in (de)hydrogenative processes* Often hemilabile (e.g., thioether), often resulting in stabilization of reactive intermediates	*Activity in (de)hydrogenative processes* Binding strength may be too strong (e.g., thiolate), inhibiting substrate coordination or slowing hydride transfer

As a matter of fact, among the transition metal complexes examined, the averagely best performing in terms of both activity and productivity are bifunctional complexes bearing mixed phosphine-thioether chelating centers, suggesting that the eventual replacement of phosphorus in homogeneous catalysts is still a remote goal to achieve, but at the same time representing a topic with a substantial room for improvement. Notwithstanding the employment of organosulfur ligands in (de)hydrogenative reactions of organic molecules is still at its dawn and requires further investigation for enhancing the enantioselection and reaching a better understanding the effect of thioether ligands in borrowing hydrogen processes, this account clearly shows their great potential and thus the upcoming role in the design of active and productive homogeneous catalysts.
